# Immune monitoring for relapse of acute myeloid leukemia after allogeneic stem cell transplantation in clinical laboratories

**DOI:** 10.3389/fimmu.2026.1778853

**Published:** 2026-04-07

**Authors:** Abdullah Demir, Furkan Aydın, Gülderen Yanıkkaya Demirel

**Affiliations:** Department of Immunology, Faculty of Medicine, Yeditepe University, Istanbul, Türkiye

**Keywords:** acute myeloid leukemia, allogeneic stem cell transplantation, clinical laboratory, immune monitoring, relapse, minimal residual disease, immunophenotyping, immune reconstitution

## Abstract

Relapse remain the leading cause of treatment failure after allogeneic hematopoietic stem cell transplantation (allo-HSCT) in patients with acute myeloid leukemia (AML), despite substantial advances in transplant strategies and supportive care. The dynamics of immune reconstitution (IR) critically determine post-transplant outcomes by shaping the balance between graft-versus-leukemia (GvL) effects, graft-versus-host disease (GvHD), infectious complications, and leukemic immune escape. Importantly, IR is not limited to numerical recovery of immune cells but represents a multidimensional and temporally organized process encompassing quantitative, qualitative, and functional immune restoration. In this review, we provide an integrated clinical laboratory–oriented framework for immune monitoring (IM) after allo-HSCT, with a specific focus on relapse prediction and risk stratification in AML. We discuss the sequential kinetics of innate and adaptive immune recovery, key cellular subsets influencing GvL efficacy, and the impact of transplant-related factors, immunosuppression, and viral reactivations on IR trajectories. Particular emphasis is placed on functional immune states, including T-cell exhaustion, anergy, and senescence, as measurable laboratory correlates of impaired immune surveillance and impending relapse. We further outline current IM methodologies used in routine and advanced clinical laboratories, including multiparameter flow cytometry, measurable residual disease (MRD) assessment, immune repertoire analysis, and emerging omics-based approaches. By integrating immunophenotypic, molecular, and functional data, IM enables earlier detection of relapse-associated immune dysfunction and supports preemptive, risk-adapted therapeutic interventions such as donor lymphocyte infusion or immunomodulatory strategies. Overall, this review highlights the pivotal role of comprehensive, longitudinal immune monitoring in translating complex immunological data into clinically actionable insights. Expanding IM beyond conventional parameters toward integrated, multidimensional approaches is essential for improving relapse prediction, personalizing post-transplant management, and ultimately enhancing long-term outcomes in AML patients undergoing allo-HSCT.

## 1 Introduction

### 1.1 Core concepts of immune reconstitution

**Figure 1:**
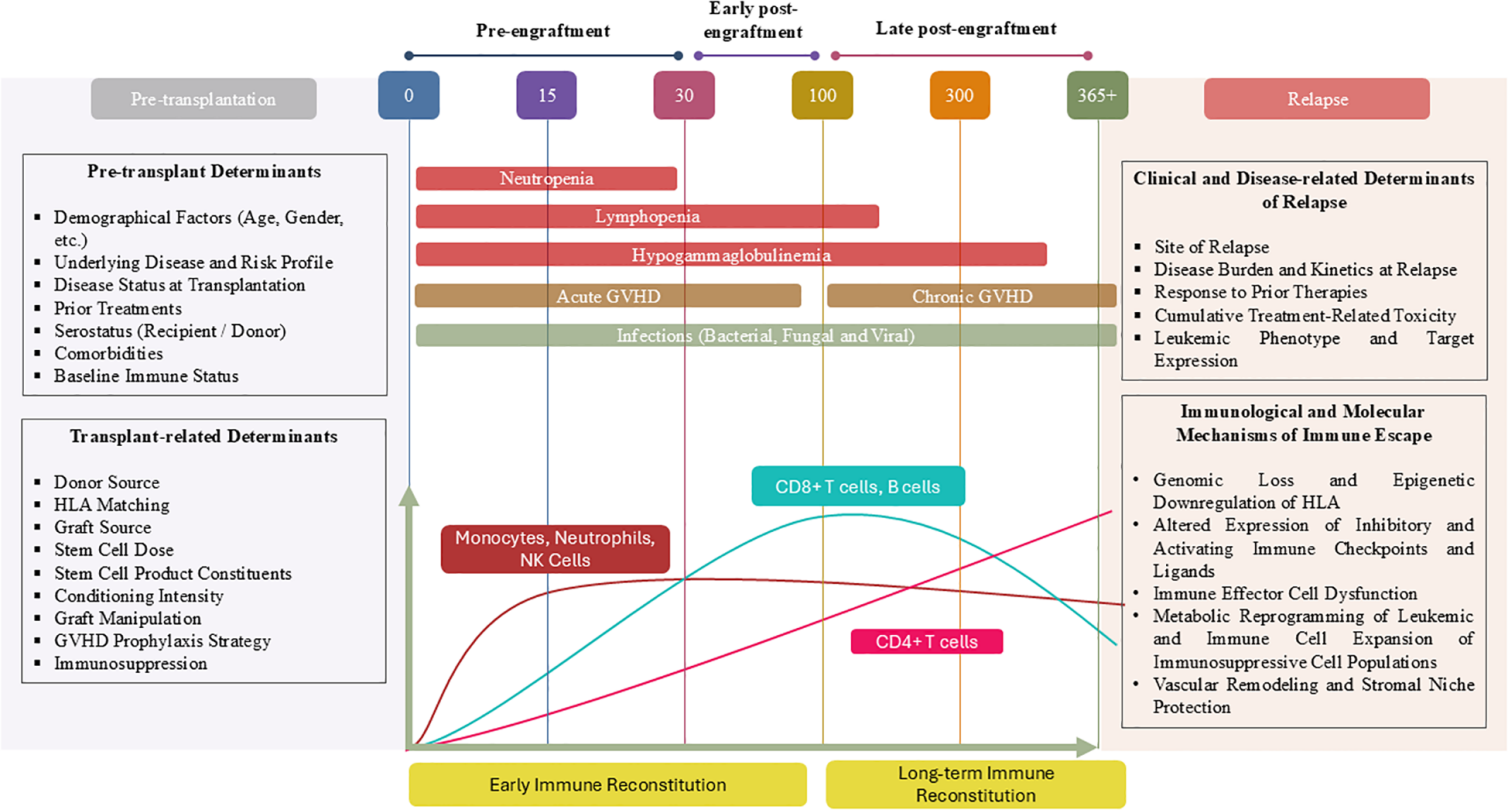
Integrated conceptual framework of IR kinetics, clinical complications, and mechanisms of leukemic immune escape following allo-HSCT. This figure presents a schematic overview of the temporal organization of IR following allo-HSCT, illustrating how sequential phases of immune recovery intersect with post-transplant complications and leukemia relapse. IR is depicted as a dynamic and multidimensional process rather than a simple numerical normalization of immune cell counts, encompassing coordinated quantitative, qualitative, and functional immune recovery over time (7). In parallel, failure of effective immune surveillance contributes to leukemia relapse through the emergence of immune escape mechanisms, including impaired antigen presentation and immune evasion pathways (8). As immune recovery progresses, the restoration of immune competence is shaped by systemic and functional immune regulation, extending beyond cell numbers to include immune repertoire diversity and effector capacity (9). These qualitative and functional dimensions are increasingly recognized as central determinants of post-transplant outcomes and long-term immune homeostasis (10). In the early post-transplant period, profound immune deficiency is associated with a heightened risk of infections and acute graft-versus-host disease, while innate immune compartments represent the first components to reconstitute and provide partial immune protection (11). Adaptive immune compartments recover more slowly and asynchronously, extending into the late post-transplant period and defining long-term immune competence. Superimposed on these processes, treatment-related factors and sustained immune pressure promote immune dysfunction and leukemic immune escape, ultimately contributing to disease relapse (12). By integrating immune recovery kinetics with clinical events and immune escape pathways within a unified temporal framework, this figure provides a conceptual basis for longitudinal, laboratory-based immune monitoring and risk-adapted intervention strategies after allo-HSCT (13, 14). GVHD, graft versus host disease; HLA, Human Leukocyte Antigen; NK, Natural Killer.

Allogeneic hematopoietic stem cell transplantation (allo-HSCT) stands as a potentially curative therapy for a range of high-risk hematological malignancies including acute myeloid leukemia (AML) (1). The success of this procedure is fundamentally dependent on immune reconstitution (IR), the complex biological process by which a functional, donor-derived immune system gradually repopulates the recipient eventually maintaining immune homeostasis (2, 3) From a clinical and biological standpoint, IR extends far beyond the mere numerical recovery of circulating immune subsets. Instead, it encompasses the coordinated restoration of immune cell abundance, phenotypic composition, repertoire diversity, and functional competence, all of which evolve over time and collectively determine clinical outcomes (4, 5). This process is of central importance, as its trajectory governs the precarious equilibrium between necessary allo-reactivity and pathological alloreactivity. On one hand, a robustly reconstituting immune system mediates the critical Graft-versus-Leukemia (GvL) effect essential for eradicating residual malignant cells (6). On the other hand, as a major predictor of post-transplant failure, a dysregulated or delayed IR can lead to severe complications, including life-threatening infections, disease relapse, and the damaging alloreactive response known as Graft-versus-Host Disease (GvHD) (**Figure 1**) (15).

From a quantitative perspective, IR refers to the recovery of absolute immune cell counts in peripheral blood and tissues, including innate and adaptive immune compartments. While this dimension provides an essential baseline for clinical monitoring, numerical recovery alone is insufficient to reflect immune effectiveness (13). Patients may exhibit apparently adequate cell counts while remaining highly susceptible to infections, relapse, or immune-mediated complications, underscoring the limitations of purely quantitative assessment (16, 17). The qualitative dimension of IR captures the phenotypic and developmental characteristics of the recovering immune system. Following transplantation, immune recovery is frequently skewed toward immature, activated, or memory-biased cell populations, reflecting peripheral expansion of donor-derived cells rather than *de novo* lymphopoiesis (18, 19). Alterations in subset distribution, delayed thymic output, restricted T-cell receptor (TCR) and B-cell receptor (BCR) diversity, and imbalanced regulatory networks are hallmarks of incomplete or distorted qualitative reconstitution and may persist long after hematologic engraftment (20). Functional IR represents the final and clinically most relevant dimension. It refers to the ability of immune cells to execute effective effector responses, including cytotoxic activity, cytokine production, antigen presentation, immune regulation, and tolerance induction (21–24). Functional recovery is shaped by conditioning-related tissue damage, inflammatory signaling, viral reactivations, and ongoing immunosuppressive therapy, resulting in immune responses that may be quantitatively present but biologically impaired (25–27). Importantly, functional competence determines the capacity to control opportunistic infections, prevent GvHD, and sustain GvL surveillance and long-term disease control. Taken together, IR should be regarded as a temporally structured and biologically integrated process rather than a single clinical milestone (28). The quantitative, qualitative, and functional dimensions of immune recovery are interdependent but not synchronous, and discordance among them is common in the post-transplant setting. This conceptual framework provides the foundation for interpreting immune monitoring (IM) data, understanding interindividual variability in post-transplant outcomes, and designing intervention strategies aimed at selectively enhancing protective immunity while minimizing transplant-related toxicity (13, 29–31).

The immune restoration following allo-HSCT proceeds through sequential, temporally asymmetric phases, transitioning from a state of profound immune compromise to the eventual establishment of a balanced, donor-derived immune system (32, 33). This process is conventionally divided into early, intermediate, and late phases, defined by the specific kinetics of myeloid and lymphoid recovery. Immediately following the conditioning-induced nadir, the innate immune system is the first to achieve numerical recovery (14). Neutrophils and monocytes typically normalize within the first 2 to 3 weeks post-transplantation. Interestingly, total peripheral blood monocyte counts often normalize earlier than neutrophil and platelet counts. This early myeloid recovery is critical, as it has been shown to be associated with the subsequent success of CD4^+^ T-cell reconstitution (34).

Natural Killer (NK) cells (CD3^neg^CD56^+^) constitute the first wave of lymphoid reconstitution, frequently attaining steady-state concentrations (≥150/μL) between the first and fourth months post-transplant (35). During this phase, the NK cell compartment is predominated by the immature, regulatory CD56^bright^ subset, which subsequently differentiates into the mature, cytotoxic CD56^dim^ phenotype. Robust NK cell recovery during this window is associated with significantly improved overall survival (OS) and a reduction in non-relapse mortality (NRM) (36). As potent innate effectors, NK cells are capable of mediating GvL activity without inducing significant alloreactivity or GVHD particularly in settings of killer-cell immunoglobulin-like receptor (KIR) mismatch or haploidentical transplantation. However, a disrupted reconstitution pattern, characterized by delayed expansion and a significant reduction in the CD56^bright^ subset, has been correlated with increased risk of occurrence of acute GvHD (aGvHD) (37).

T-cell reconstitution is a prolonged process dictated by thymic function, central to GvL and long-term protection. Recovery proceeds via early cytokine-driven peripheral expansion of mature donor T cells, followed by sustained thymic output (*de novo* thymopoiesis) of naïve T cells (38). The recovery of the T-cell compartment follows two distinct and sequential pathways; peripheral expansion, in the early post-transplant weeks, mature immunocompetent T cells residing within the donor graft undergo cytokine-driven clonal expansion (39, 40). This initial expansion provides rapid, albeit limited, immunity. And thymic output (*de novo* thymopoiesis), long-term, high-quality recovery, relies on hematopoietic stem cells differentiating into naïve T cells that require education in the recipient’s thymus. This *de novo* thymopoiesis is essential for building a diverse T-cell repertoire and achieving self-tolerance. CD8^+^ T cells often recover numerically within months, whereas CD4^+^ T-cell reconstitution may take years (41, 42).

Impaired thymopoiesis, often resulting from the conditioning regimen, increasing host age, or GvHD, contributes significantly to prolonged T-cell incompetence and delayed recovery (43). The recovery of the CD4^+^ T-cell subset is a crucial prognostic marker and the main predictor for long-term immunity, particularly against viral pathogens such as cytomegalovirus (CMV) (44). Failure to achieve adequate early CD4^+^ counts, such as meeting a threshold of ≥50 cells/μL in the first 100 days, indicates an increased risk of complications, establishing a critical “window of vulnerability”. Conversely, the B-cell compartment demonstrates the most protracted recovery course among all major lymphocyte subsets, often failing to reconstitute fully until years after allo-HSCT (45, 46). Humoral immunity is restored slowly, with IgG2 levels typically remaining low for prolonged periods, increasing the risk of late bacterial infections. Achieving coupled CD4 and B-cell IR (B cells>25 cells/μL before day 100) is associated with substantially decreased NRM, aGVHD, and a reduced risk of relapse specifically in the AML subgroup (47).

The rate and quality of IR are influenced by a multitude of clinical and transplant factors that necessitate a shift toward dynamic, risk-adapted monitoring algorithms (48). Key influencing factors include the conditioning regimen, the stem cell source, and strategies for T-cell depletion (49). Immunosuppressive therapy agents and GvHD itself delay or skew lymphoid recovery (50). The use of post-transplant cyclophosphamide (PTCy) as GvHD prophylaxis actively modulates the IR trajectory by supporting rapid regulatory T cell (Treg) reconstitution, which effectively reduces rates of aGvHD and chronic GVHD (cGvHD) without compromising the critical GvL effect (51). Furthermore, clinical events must be incorporated into IR interpretation: CMV reactivation is observed to be a strong stimulus for global T-cell reconstitution, while human herpesvirus-6 (HHV-6) reactivation may exert an opposite, suppressive effect on T-cell counts (52).

A major mechanism contributing to GvL failure is the development of T-cell exhaustion, which occurs following chronic exposure to leukemia-associated antigens. This exhaustion results in the sustained up-regulation of multiple inhibitory receptors, leading to the progressive loss of T-cell function. The clinical laboratory can detect phenotypic features consistent with this dysfunctional state by measuring the co-expression of inhibitory receptors, for example PD-1^bright^TIM-3^+^ T cells, using multi-parametric flow cytometry (8, 53). This specific phenotypic signature reliably associates with and predicts leukemia relapse in AML patients post-allo-SCT. These PD-1^bright^TIM-3^+^ T cells are demonstrably functionally deficient, exhibiting reduced production of essential effector cytokines, including interleukin-2 (IL-2), tumor necrosis factor-α (TNF-α), and interferon-γ (IFN-γ) (53). Consistently, in relapsed AML/MDS after allo-HCT, high frequencies of CTLA-4/PD-1– and multi-ICM–co-expressing T cells and blasts are detectable in uncultured blood and associate with impaired antileukemic activity and poor response to salvage therapy (54). Crucially, the increase in PD-1^bright^ TIM-3^+^ cells occurs before the clinical or morphological diagnosis of leukemia relapse, offering a critical lead time. This advanced warning allows clinicians to implement preemptive therapeutic strategies, such as Donor Lymphocyte Infusion (DLI) or immune checkpoint blockade, with the potential to reverse the exhausted state and restore GvL function before overt relapse occurs. Therefore, the assessment of T-cell functional quality via exhaustion markers is paramount and augments purely quantitative IM (55).

Understanding relapse after allo-HSCT demands more than isolated measurements — it requires tracing a coherent biological and clinical logic from fundamental mechanisms to bedside application. We therefore begin by examining the cellular foundations of IR, whose kinetics and quality determine whether GvL surveillance is effectively maintained or progressively erodes. Disruptions in this process do not occur in silence; they leave measurable immunological signatures that can be captured in routine clinical laboratories and translated into relapse risk. The challenge then becomes methodological: we survey the technological platforms — from multiparameter flow cytometry and MRD assays to omics-based approaches — that enable this translation, while critically examining their complementary strengths and practical limitations. Finally, we address what happens after the data is generated, outlining how multiparametric measurements can be integrated into structured, risk-oriented reporting frameworks and how rule-based, statistical, and AI-assisted decision systems can make these outputs actionable at the level of individual patient management.

### 1.2 Rationale for immune monitoring

The immune system is a dynamic and complex network in which numerous cellular and molecular interactions occur simultaneously in a context that is both spatially and temporally regulated (56). The meaningful interpretation of immune responses in routine clinical laboratory practice extends beyond an understanding of their biological basis and requires the reliable and sustainable implementation of monitoring strategies that enable patient stratification into distinct risk groups and support the prediction of prognosis and relapse risk (57, 58). For many years, established techniques such as flow cytometry, microscopy, and immunohistochemistry have been widely used in the diagnosis and monitoring of diseases, as well as in studies aiming to predict subsequent clinical outcomes. With advances in technology, approaches capable of simultaneously assessing multiple parameters and resolving disease heterogeneity at the single-cell level have been increasingly integrated into routine diagnostics and research applications (59). All methods used in research and integrated into routine monitoring undergo multi-step verification processes supported by validation studies, including assessments of the limit of detection (LOD), lower limit of quantification (LLOQ), linearity, precision, and specificity (60). Validating the clinical relevance of biomarkers and methods used in IM can be challenging in many respects. Careful planning and appropriate study design are therefore critical (61).

IM is a methodology that enables the phenotypic assessment of immune cells and their associated microenvironment by capturing molecular and functional relationships, and supports decision-making in clinical monitoring and therapeutic processes (62). As a field with significant potential for further development, IM serves a broad range of applications, including advanced cell therapies and transplantation processes, the evaluation of immunotherapies in immuno-oncology, as well as disease surveillance, precision medicine approaches, allergy, immunometabolism, and autoimmune disorders (56, 63–68). General IM approaches encompass functional assays, immunophenotyping, omics technologies, and spatial methods, with their applications and representative platforms summarized in **Table 1**.

**Table 1:** Overview of immune monitoring approaches in clinical and research applications.

Sample type	IM methods	Method subtypes	Applications	Platforms/Technologies	Ref.
Peripheral blood, PBMCs	Functional assays	ELISPOT/FluoroSpot (C/R), *in vitro* suppression assays (R), CD137/CD154 activation assays (R), intracellular cytokine staining proliferation assays (R), NK cytotoxicity assays (R)	Antigen-specific immune responses, immunotherapy efficacy, vaccine responses, autoimmunity, GvHD	ELISPOT readers, flow cytometry-based functional assays	(69–74)
Peripheral blood, bone marrow	Immunophenotyping (conventional)	Multicolour flow cytometry (C/R),basic lymphocyte subset analysis (C)	Patient stratification, immune status assessment, clinical monitoring	Flow cytometers (8–18 colours)	(75)
Peripheral blood, tissue	Extended immunophenotyping	High-parameter cytometry (>20 markers) (C/R),phospho-flow (R), activation and exhaustion marker panels (C/R)	Treatment response evaluation, signalling pathway analysis, functional immune states	Spectral flow cytometry systems	(76)
Peripheral blood, bone marrow	Mesurable residual disease (MRD)-Flow cytometry	Leukemia-associated immunophenotype (LAIP)/different-from-normal (DfN) approaches (C/R),high-sensitivity MRD panels (C/R)	Disease burden monitoring in hematological malignancies, prognostic assessment	High-sensitivity multicolour flow cytometry	(77)
Peripheral blood, bone marrow	MRD (molecular level)	Droplet digital PCR (ddPCR) (C/R), quantitative PCR (C/R), targeted molecular MRD assays (C/R)	Treatment response assessment, relapse risk prediction, post-transplant monitoring	ddPCR platforms, real-time PCR systems	(78)
Peripheral blood, tissue	Mass cytometry	Cytometry by Time-of-Flight (CyTOF) -based high-dimensional cellular profiling (R)	Systems-level immune mapping, translational research	CyTOF	(79)
Tissue biopsies	Histology/IHC	Conventional IHC (C), multiplex IHC (C/R)	Tissue architecture assessment, spatial immune distribution	Digital pathology systems	(80)
Blood, tissue, single-cell suspensions	Omics approaches	Bulk RNA sequencing (R), single-cell RNA sequencing (scRNA-seq) (C/R)	Mechanistic studies, biomarker discovery	Next-generation sequencing (NGS) platforms	(81)
Blood, tissue	Epigenomic analyses	ATAC-seq (R), DNA methylation profiling (C/R)	Cellular differentiation, immune memory characterisation	NGS-based epigenomic platforms	(82)
Serum, plasma	Proteomic/metabolomic profiling	Mass spectrometry (C/R), cytokine panels (C/R), immunometabolic profiling (R)	Systemic inflammation assessment, metabolic immune states	Liquid chromatography tandem-mass spectrometry (LC-MS/MS), multiplex bead-based assays	(83)
Peripheral blood, bone marrow	TCR/BCR repertoire and clonal tracking	Clonal expansion analysis (C/R), diversity metrics (R), MRD integration (C/R)	Hematological malignancies, immunotherapy, transplantation	NGS-based immune repertoire sequencing platforms	(84–86)
Tissue sections	Spatial imaging approaches	Imaging mass cytometry (IMC) (R), multiplex immunofluorescence (C/R), spatial transcriptomics (R)	Microenvironment analysis, cell-cell interaction mapping	IMC systems, digital imaging platforms	(87)
Serum, plasma	Soluble immune biomarkers	Cytokines, chemokines, soluble checkpoint molecules (C/R)	Therapy response monitoring, toxicity assessment, inflammation profiling	Multiplex immunoassays	(88)
Clinical and research data	Computational and digital immunology	Artificial intelligence (AI) (R), machine learning (ML) (R), predictive modelling (R)	Risk stratification, patient classification	AI/ML-based analytical frameworks	(89)

C, Clinical monitoring methods; R, Research monitoring methods; C/R, Monitoring methods used in both clinical and research settings.

IM has been substantially advanced through the adoption of high-parameter cytometry and omics-based technologies (90). Beyond the detailed insights offered by multi-parametric indicators, measurements of cell number, cell size, and cell-to-cell ratios provide practical advantages for IM in specific clinical and research settings (90). World Health Organization (WHO) reports indicate that more than 90% of healthcare facilities worldwide are able to perform complete blood count testing rapidly. In this context, composite indices that integrate ratios of multidimensional parameter, such as neutrophils, monocytes, platelets, and lymphocytes, may offer greater clinical value than individual hemogram parameters alone (91). Within this framework, simple ratios such as the neutrophil-to-lymphocyte ratio have become well-established, practical, and readily accessible biomarkers across diverse disease settings (92). These indices based on cell count ratios provide prognostically important information, even in diseases with challenging clinical management such as AML. Lymphocyte-to-monocyte ratio (LMR) and monocyte-to-lymphocyte ratio (MLR) are among the cell-to-cell metrics proposed to reflect the myeloid–lymphoid balance and to indicate early IR in the post–stem cell transplantation setting. Available studies suggest that early reversal of LMR (LMRR) following allo-HSCT may be associated with a lower risk of relapse and improved survival outcomes (93). The platelet-to-lymphocyte ratio (PLR), particularly in the pre-transplant or early post-transplant period, is considered a composite marker reflecting both systemic inflammation and the underlying hematopoietic and immune status (94). Studies reporting an association between the pre-transplant CD4/CD8 ratio and post-transplant outcomes, such as relapse risk, consider CD4/CD8 dynamics to be of interest within the broader framework of IR monitoring (95, 96).

The identification and monitoring of complex immune cell fate decisions within IM frameworks represent an area of growing interest for both research and clinical practice. In this context, discriminating between phenotypes related to cellular exhaustion, anergy, and immunosenescence, and accurately defining cell fate, is considered critical for evaluating predictive and prognostic factors associated with responses to immunotherapy (97, 98). Cell exhaustion is generally defined as the impairment or loss of a cell’s functions. However, researchers have proposed that cellular exhaustion should be viewed not merely as a loss of function, but also as an active and coordinated differentiation program (99). In contrast to exhaustion, anergy refers to the functional silencing of immune cells at an earlier stage, typically during the priming phase. This process is centrally regulated by transcriptional and post-translational mechanisms, with early growth response 2 (Egr2) and E3 ubiquitin ligases, including casitas B-lineage lymphoma-b (Cbl-b), gene related to anergy in lymphocytes (GRAIL), and Itch, playing key roles (98). Senescence represents a permanent state of cell cycle growth arrest, induced by various stressors including telomeric attrition and oncogenic signaling (100). The biological trajectories underlying exhaustion and senescence represent distinct cell fate programs. One of the most critical observations in IM is that, in human CD8^+^ T cells, the expression of killer cell lectin-like receptor G1 (KLRG1) and programmed cell death protein 1 (PD-1) is typically inversely correlated (101). Recent studies indicate that AML cells drive NK cell exhaustion by upregulating the NKG2A/HLA-E axis and suppressing the PI3K/AKT pathway, thereby facilitating relapse following allo-HSCT (102). Improving post-allo-HSCT outcomes requires a strategic balance: suppressing Graft-versus-Host Disease GvHD without compromising GvL efficacy. Current approaches range from T-cell depletion to engineering T-cells that target minor histocompatibility antigens (miHAs). However, leukemic escape remains a critical challenge. Post-transplant relapse is often driven by the downregulation of host miHAs and the functional exhaustion of miHA-specific CD8 T-cells characterized by the upregulation of inhibitory markers like PD-1 and Tim-3, which ultimately leads to GvL resistance (103–105). Additionally, T-cell anergy can be induced via the activation of TIM3 by its ligand, Galectin-9, which is overexpressed on AML blasts. This interaction triggers several downstream signaling pathways, including mitogen-activated protein kinase (MAPK)/extracellular signal-regulated kinase (ERK), PI3K, and AKT, further promoting immune evasion (106). Moreover, cellular senescence acts as a key factor facilitating relapse in AML (107) in AML patients undergoing chemotherapy, elevated pretreatment frequencies of CD28^neg^CD57^+^CD8^+^ T cells are associated with poor clinical outcomes. This senescent T-cell phenotype serves as a predictor for significantly reduced OS and event-free survival (EFS) (108). These findings indicate that the fate of immune cells, specifically through senescence, anergy, and exhaustion, is intimately linked to AML relapse. In routine clinical laboratories, comprehensive assessment of cell fate states such as exhaustion, anergy, and senescence can be technically challenging and resource-intensive. However, in pre- and post-transplant follow-up samples, the use of optimized multicolor immunofluorescence panels (OMIPs) and standardized flow cytometry panels may facilitate IM of these cellular states (109–111). Stratifying transplant recipients into defined subgroups and evaluating phenotypes associated with anergy, exhaustion, and senescence may help practically identify IM parameters with potential predictive and prognostic relevance.

Clinical planning and immunological monitoring are becoming closely linked, especially in fields like oncology, transplantation, and infectious diseases. Although more refined methodologies continue to be developed, several immune parameters and monitoring approaches already exert a direct influence on clinical decision. In clinical practice, IM plays a critical role in a wide range of decision-making processes, including the postponement of primary vaccination, determination of booster dose initiation, adjustment of immunosuppressive dosing or timing, selection of the optimal onset of immunotherapy, scheduling of chimeric antigen receptor T (CAR-T) cell or other adoptive cell therapy infusions, and the timing of vaccination following rituximab treatment or B-cell depletion. Current guidelines recommend initiating vaccination 3–6 months after HSCT, following IR. However, vaccination timing should be individualized by taking into account factors such as the presence of GvHD and ongoing immunosuppressive therapy (112–114). Suggested criteria include a CD4^+^ T-cell count greater than 0.2 × 10^9^/L and a CD19^+^ or CD20^+^ B-cell count exceeding 0.2 × 10^9^/L (115). In patients with severe GvHD or lymphopenia (absolute lymphocyte count <500/μL or CD4^+^ T-cell count <200/μL), a booster dose may be administered as a second dose at least 4 weeks after the initial vaccination (116). TCRαβ depleted transplants delay IR in recipients of hematopoietic cell transplantation. For this reason, in TCRαβ-depleted transplants, IM of T-cell subsets provides clinicians with guidance for clinical interventions (117, 118). In this context, vaccination decisions require documented evidence of IR prior to immunization.

HSCT represents the earliest form of cell-based therapy and continues to serve as a cornerstone in the management of hematologic malignancies (119). HSCT refers to the replacement of the hematopoietic system using the patient’s own hematopoietic stem cells (autologous HSCT, auto-HSCT) or hematopoietic stem cells obtained from another individual (allo-HSCT). Currently, auto-HSCT is primarily used to restore hematopoietic function following high-dose chemotherapy in the treatment of selected hematologic and non-hematologic malignancies. Allo-HSCT is employed to treat congenital or acquired bone marrow failure syndromes and, more commonly, to exploit the graft-versus-tumor effect of allogeneic immune cells in patients with high-risk hematologic malignancies (120). Although the efficacy and safety of the HSCT procedure have improved substantially in recent years, a significant proportion of patients still experience serious complications, including severe aGvHD or cGvHD, relapse in the setting of malignant disease, acute toxicity, and viral reactivation. The factors influencing HSCT outcomes are numerous and heterogeneous, encompassing the choice of graft source, the composition and doses of infused cellular subsets, conditioning regimens, immunosuppressive strategies, and supportive care approaches. Within this complex and highly variable framework, the implementation of systematic IM before and after transplantation is of critical importance for assessing IR and for the early prediction of complication risks (30). In the pre-transplant period, standard IM assays are performed in clinical laboratories to assess patients’ immune competence, with routine flow cytometry-based immunophenotyping panels forming the foundation of this evaluation (121). In the pre-transplant setting, in addition to immunophenotypic assessments, a range of molecular assays may be performed to characterize leukemogenesis at the genomic level. Beyond routine immunophenotyping and mutation analyses, the use of additional immunomolecular monitoring approaches is left to the discretion of the clinician (122). In allo-HSCT, the presence of donor-specific anti-HLA antibodies (DSA) in the recipient is strongly associated with delayed engraftment and primary graft failure. Therefore, particularly in HLA-mismatched and haploidentical transplantations, pre-transplant HLA antibody screening for the identification of DSA and the monitoring of desensitization therapies in histocompatibility laboratories are considered essential components of the donor selection process (123). Flow cytometry is a powerful tool for the assessment of immunophenotypic characterization in the post-HSCT period. Using routine panel analyses, disease-specific immunophenotypic monitoring can be performed across different malignancies (124, 125). In the post-transplant setting, MRD monitoring can be performed using molecular techniques such as ddPCR and NGS in addition to flow cytometry. Monitoring MRD levels provides a powerful predictive approach for assessing the risk of relapse (126, 127). Approaches to monitoring IR may vary according to the technical and financial resources of individual centers, as well as clinicians’ therapeutic and follow-up strategies. Nevertheless, systematic assessment of the key immune cell subsets summarized in section 1.1 during the pre- and post-transplant period may strengthen prognostic IM and provide a rational framework for immune surveillance that has the potential to improve long-term survival outcomes. At this stage, rather than limiting immunophenotypic characterization to restricted, diagnosis-driven single-panel analyses, the use of comprehensive immunophenotyping panels that allow simultaneous assessment of myeloid cells, T and B lymphocytes, NK cells, and their activation, precursor, and maturation states enables a more holistic and clinically meaningful evaluation of post-transplant IM. When clinically indicated, deep immunophenotyping approaches may further provide a more detailed and functional characterization of IR. Such integrated IM strategies are pivotal for effectively translating laboratory-derived data into clinical decision-making, thereby playing a critical role in the realization of the “from bench to bedside” paradigm (128).

## 2 Graft-versus-host disease and immune monitoring

**Figure 2:**
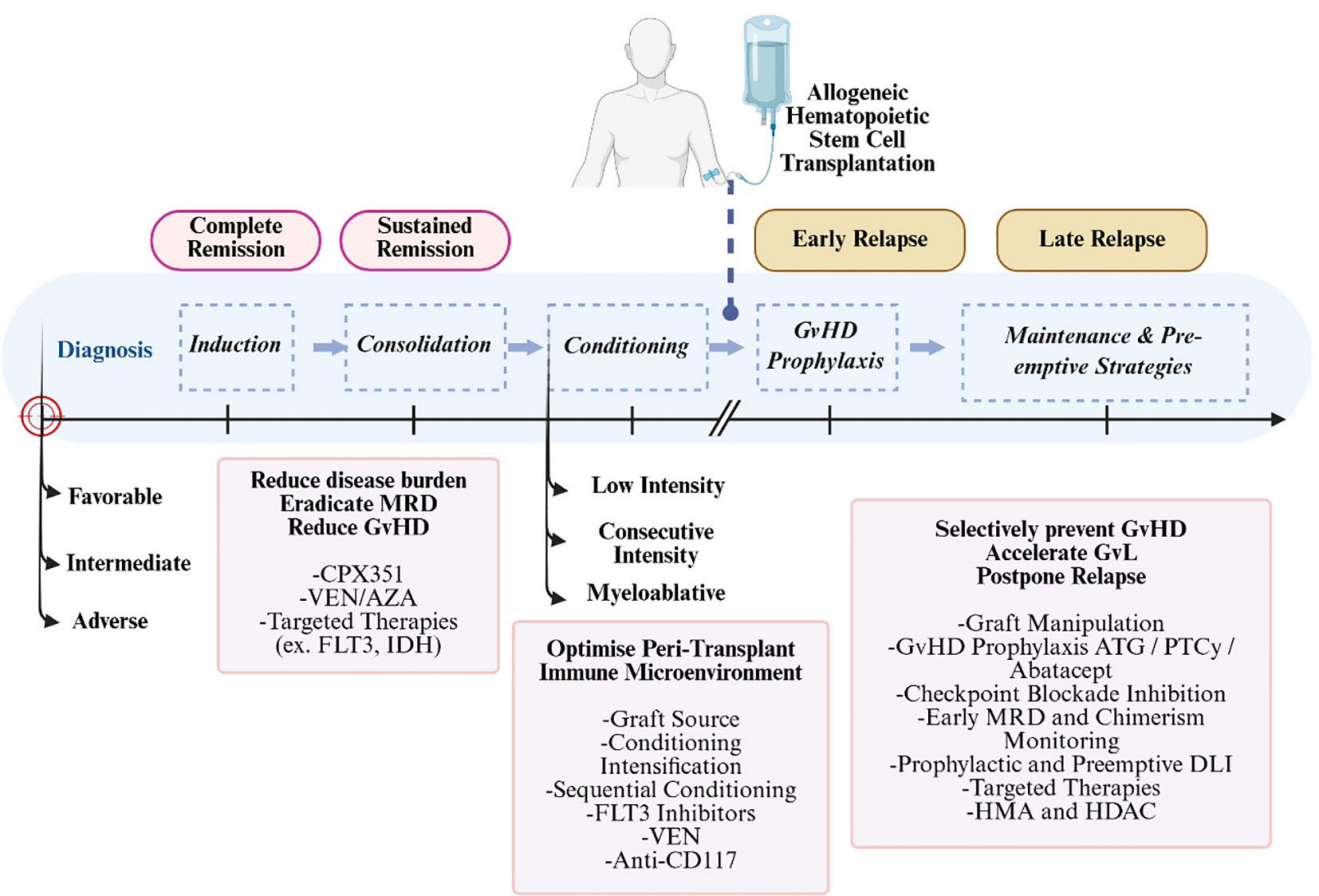
The Dynamic Therapeutic Ecosystem of AML: From Molecular Priming to Donor-Mediated Immune Surveillance. The clinical management of AML is delineated as an integrated continuum of pharmacological and immunological interventions (138). Pre-Transplant Molecular Priming: Induction and consolidation with modern agents (e.g., Venetoclax, FLT3/IDH inhibitors, CPX-351) aim for maximal cytoreduction and deep MRD eradication (139). Beyond debulking, these targeted therapies exert an “imprinting” effect on the host environment; for instance, FLT3 inhibitors like Sorafenib/Gilteritinib prime the immunological niche by increasing IL-15 production and downregulating co-inhibitory receptors (e.g., PD-1, TIGIT), thereby optimizing subsequent T-cell and NK-cell dynamics (10, 140). Conditioning and Microenvironment Optimization: Selection of conditioning intensity (MAC *vs*. RIC) is guided by a balance between disease aggressiveness (defined by MRD depth and ELN 2022 risk) and recipient fitness (141). Achieving deep pre-transplant molecular remissions may permit the use of lower-intensity conditioning without compromising the Graft-versus-Leukemia (GvL) effect (138). GvHD Prophylaxis and GvL Genesis: Post-transplant platforms, particularly PTCy and Abatacept, facilitate selective alloreactive T-cell depletion while sparing memory and regulatory T cells, essential for fostering a “homeostatic” immune recovery and GvL initiation (142). Maintenance and Pre-emptive Surveillance: Longitudinal monitoring of MRD (via NGS/Flow) and donor chimerism serves as a trigger for pre-emptive strategies, including Donor Lymphocyte Infusions (DLI), Oral-AZA, or targeted inhibitors (139)These interventions aim to accelerate GvL kinetics to “buy time” against subclinical clones, ultimately postponing or preventing morphological relapse (10, 141). [Created in BioRender. Aydın, F. (2026) https://BioRender.com/j0lgptz].

In allo-HCT, incompatibility in HLA antigens enables donor immune cells to target and eliminate malignant hematopoietic cells, thereby mediating the GvL effect and supporting hematopoietic reconstitution. However, when these immune responses extend to healthy recipient tissues, they give rise to GVHD, one of the most severe complications and a leading cause of transplant-related mortality following allo-HCT (129). Although numerous parameters are considered during transplant planning, the choice of strategy aimed at preventing graft-versus-host disease is among the most important and modifiable factors directly influencing the likelihood of GVHD development. Each prophylactic approach entails a distinct balance of risks and benefits with respect to GVHD incidence and severity, the pace and quality of immune reconstitution, susceptibility to infections, preservation of graft-versus-tumor effects, and the occurrence of treatment-related adverse events (130). Globally accepted clinical guidelines for graft-versus-host disease (GVHD) prophylaxis are developed and regularly updated by the European Society for Blood and Marrow Transplantation (EBMT), the National Comprehensive Cancer Network (NCCN), and the American Society for Transplantation and Cellular Therapy (ASTCT). These guidelines not only outline recommended GVHD prophylaxis strategies according to donor type and transplantation platform but also emphasize the impact of these approaches on immune reconstitution and infection risk. Accordingly, the relevant prophylactic strategies are summarized in **Table 2**, together with their clinical and biological implications for immune monitoring (**Figure 2**) (143–145).

**Table 2:** Overview of relevant GVHD prophylactic strategies and their associated clinical and biological implications for immune monitoring.

Prophylaxis regimen	Immune reconstitution profile	Immune monitoring	Key AML relapse-related considerations	Ref
Calcineurin inhibitor (CNI)–based(Cyclosporine A or Tacrolimus ± MTX/MMF)	Quantitative T-cell recovery may occur, but effector function, proliferative capacity, and TCR diversity are often impaired	CD4^+^/CD8^+^ T-cell kinetics; cytokine profiles	GvL activity is generally preserved; however, prolonged and profound immunosuppression may indirectly influence relapse and infection risk	(6, 131)
Post-transplant cyclophosphamide (PT-Cy)	Adaptive α/β T-cell reconstitution is delayed, whereas NK cells and innate immune responses recover earlier	T-cell subset dynamics; TCR repertoire analysis; Treg kinetics	In AML, effective GVHD control is achieved without a consistent increase in relapse risk; GvL effects are largely preserved, although they may be attenuated early post-transplant	(132–134)
Selective α/β T-cell and CD19^+^ B-cell depletion by CliniMACS	Rapid innate and innate-like immune recovery (NK and γ/δ T cells); delayed but more balanced and physiologic adaptive α/β T-cell reconstitution	Lymphocyte subset analysis	Allows higher infused cell doses while preserving the balance between GVL efficacy and reduced GVHD risk.	(118, 135, 136)
Anti-thymocyte globulin (ATG)	The kinetics of immune cell subpopulation reconstitution (delayed for ATG-treated CD4^+^ T cells; rapid for NK and B cells)	Lymphocyte subset analysis	The reduction of GVHD by ATG accompanied by delayed CD4^+^ T-cell reconstitution and its potential impact on the GVL/relapse balance	(137)

ATG, Anti-thymocyte globulin; CNI, Calcineurin inhibitor; CsA, Cyclosporine A; EBV, Epstein–Barr virus, IL-2, Interleukin-2; MMF, Mycophenolate mofetil; MRD, Minimal residual disease; MTX; Methotrexate; NFAT, Nuclear factor of activated T cells, PT-Cy, Post-transplant cyclophosphamide; PTLD, Post-transplant lymphoproliferative disorder; TCR, T-cell receptor; Teff, Effector T cell; Treg, Regulatory T cell.

## 3 Immune monitoring of AML relapse with immunophenotyping

Flow cytometry, with its capacity for multiparametric analysis, provides rapid and highly sensitive immunophenotyping and represents a cornerstone laboratory tool for both the identification of therapeutic targets and post-treatment IM. The WHO-HAEM5 and ICC classifications incorporate immunophenotypic features into the diagnostic criteria for AML, alongside cytogenetic and molecular findings (146, 147). In this context, flow cytometry is indispensable for the detection and quantification of leukemic blast populations, lineage assignment, and the identification of aberrant immunophenotypic patterns. These features are critical not only for the accurate diagnosis and subclassification of acute myeloid leukemia, but also for the assessment of post-transplant IR and the monitoring of MRD following therapy (124).

In immunophenotyping, the major challenges arise from inter-laboratory variability in instrumentation, antibody panel design, data analysis approaches, and reporting formats, which collectively hinder standardization and the reliable comparison of generated data. To address these challenges, the implementation of well-defined SOPs and the adoption of standardized data analysis workflows are of critical importance, ensuring reproducibility and consistency across laboratories (148). In this context, the development of shared and standardized monitoring protocols is essential to enable meaningful correlation of laboratory outputs with different therapeutic strategies. In AML reference frameworks for the standardization, validation, and harmonization of flow cytometry-based approaches are provided by clinical guideline bodies such as the European Leukemia Network (ELN) and the National Comprehensive Cancer Network (NCCN), complemented by methodological and technical recommendations from the International Clinical Cytometry Society (ICCS). In addition, multicenter collaborative initiatives such as the HARMONY Alliance play an important role in the validation of methodologies for MRD assessment and in establishing their clinical relevance (see section 1.3) (149–152).

At our center, in patients diagnosed with AML, we perform comprehensive IM using an extensive flow cytometry panel that encompasses the activation, maturation, and progenitor subpopulations of myeloid cells, both at initial diagnosis and during post-treatment follow-up (**Table 3**). In addition to detailed immunophenotypic characterization of myeloid cells, T and B lymphocytes as well as NK cells are analyzed concurrently, allowing for an integrated evaluation of both disease-specific features and the host immune system. Additionally, our assessments include the evaluation of cell activation markers, as well as cells within the platelet and erythroid lineages. This expanded panel approach not only confirms the preliminary diagnosis provided by the clinician but also enables the detection of possible mixed-phenotype acute leukemia (MPAL), thereby enhancing diagnostic accuracy and directly contributing to the formulation of the treatment strategy. Routine lymphocyte subset analysis in every sample facilitates the dynamic monitoring of IR, particularly in the post-transplant period. Moreover, our comprehensive immunophenotyping strategy also allows for the early identification of phenotypic shifts or potential disease transformations that may occur during the post-treatment phase., evaluation with an integrated panel enables the detection of potential aberrant expressions, thereby guiding clinicians in the optimization of their therapeutic strategies Through this integrated IM approach, we provide the clinician with multidimensional, clinically meaningful data that extend beyond diagnosis and risk stratification to include treatment planning, assessment of response to therapy, and insights into the long-term course of the disease. Systematic immunophenotyping analyses are performed at 15, 30, 60, and 90 days, as well as at 6 months and 1 year post-transplantation. For patients remaining in remission throughout the first year, the follow-up process is sustained through periodic immunophenotypic examinations of bone marrow aspiration samples, conducted at intervals (3–6 months) determined by the clinician.

**Table 3:** Flow cytometry panels utilized in our clinical laboratory for initial diagnosis, remission monitoring, and relapse follow-up.

Panel	FITC	PE	ECD	PC5.5	PC7	APC	ALEXA 700	ALEXA 750	PACIFIC BLUE	KROME ORANGE
1st Panel	CD15	CD33	CD13	CD117	CD34		CD56	CD16	CD14	CD45
2nd Panel	CD3		CD4	CD41	CD8	CD2	CD7	CD71		CD45
3rd Panel	CD36	CD10	CD24	CD19	CD34	HLA-DR	CD22	CD38	CD20	CD45
4th Panel	TdT	cMPO				cCD79a		cCD3		

Gray shading denotes empty channels where no fluorochrome-conjugated antibody was included in the panel.

Impairments in T-cell reconstitution or function following allo-HSCT may predispose patients to immunopathological complications and disease progression. In the early post-transplant period, reconstitution of the T-cell compartment occurs predominantly through homeostatic proliferation of donor-derived mature T cells infused with the stem cell graft. This process is referred to as the thymus-independent pathway, distinguishing it from the thymus-dependent pathway, which involves *de novo* generation of naive T cells from donor-derived progenitor stem cells. Although homeostatic proliferation provides a rapid means of replenishing a severely depleted T-cell repertoire, the quantitative and qualitative characteristics of T cells generated through this mechanism are more limited compared with those produced via thymus-mediated *de novo* T-cell development. Indeed, T cells arising from donor-derived mature T cells have been shown to confer less effective protection against infectious pathogens, likely due to their restricted TCR diversity (6, 153). To elucidate the biological and clinical significance of high-level and atypical T-cell expansions observed during post-transplant monitoring, we extend our assessment of cellular immunity by performing in-depth TCR repertoire analysis. Using the panel detailed in **Table 4**, we characterize T-cell clonality based on TCRαβ and TCRγδ expression patterns. This enables a comprehensive immunophenotypic evaluation of the quality of T-cell IR, repertoire diversity, and potential lymphoproliferative processes. This strategy facilitates the distinction between reactive and truly clonal expansions, thereby providing meaningful contributions to clinical follow-up and risk stratification.

**Table 4:** Flow cytometry panel utilized for TCR repertoire assessment following allo-HSCT in our clinical laboratory.

Panel	FITC	PE	ECD	PC5.5	PC7	APC	ALEXA 700	ALEXA 750	PACIFIC BLUE	KROME ORANGE
TCRαβ/TCRγδ Panel	TCRγδ	TCRαβ	CD4		CD8			CD3		CD45

Gray shading denotes empty channels where no fluorochrome-conjugated antibody was included in the panel.

In flow cytometry-based immunophenotypic assessments, identifying a LAIP at diagnosis is a critical step in AML. This baseline phenotype serves as a key reference for monitoring MRD during both post-treatment and post-transplant follow-up. Nevertheless, AML cells are highly dynamic and show significant phenotypic variability depending on disease biology and differentiation status (154). Therefore, while tracking the initial LAIP is valuable, it should not be considered a fixed standard for evaluating disease progression. The ELN guidelines recommend a complementary approach that combines LAIP with DfN strategies to maximize the strengths of both. LAIP monitoring follows the aberrant phenotype defined at diagnosis, whereas DfN identifies abnormal antigen expression patterns that are not normally present in hematopoietic progenitors during follow-up. LAIP offers high specificity but may miss emerging immunophenotypic changes and cannot be applied when no distinct LAIP is present at diagnosis. On the other hand, DfN can detect newly appearing or evolving phenotypes but carries a risk of false positives, as some abnormalities may reflect normal regenerative bone marrow patterns or low-level expression in healthy tissue (155).

**Figure 3:**
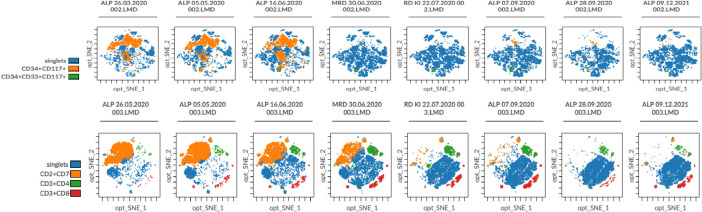
t-SNE-based dimensionality reduction visualizations of multiparameter flow cytometry data obtained from post-transplant bone marrow aspirates during remission assessment in an acute leukemia case with MPAL. The displayed plots represent high-dimensional immunophenotypic data generated using a 28-marker acute leukemia flow cytometry panel, projected into two dimensions to preserve phenotypic similarity between individual cells. Each dot corresponds to a single cell, while spatial proximity reflects immunophenotypic relatedness rather than absolute cell frequency. The opt-SNE1 and opt-SNE2 axes represent the first and second dimensions of the nonlinear embedding and do not correspond to specific biological parameters. At diagnosis, both myeloid and T-lymphoid blast populations are identified as distinct clusters. Longitudinal analysis across eight sequential time points demonstrates an earlier clearance of the myeloid blast compartment, with immunophenotypic remission achieved by the fourth time point, whereas T-lymphoid blasts exhibit delayed clearance, reaching remission at the fifth time point. Beyond blast tracking, this visualization simultaneously captures immune reconstitution dynamics, allowing the parallel assessment of CD3^+^CD4^+^ T helper cells and CD3^+^CD8^+^ cytotoxic T lymphocytes alongside leukemic populations. By integrating blast kinetics with immune subset remodeling, t-SNE-based visualization provides a compact yet information-rich framework for longitudinal monitoring of remission and relapse processes in the post-transplant setting. All visualizations were generated using the CytoBank platform (https://premium.cytobank.org/) at the Yeditepe University Hospitals Stem Cell Laboratory.

Relying solely on written reports for the longitudinal monitoring of immunophenotypic data presents a practical challenge for clinicians. In particular, in complex cases such as MPAL, the retrospective tracking of baseline-established LAIPs becomes increasingly difficult over extended follow-up periods. The implementation of an integrated platform capable of visualizing immunophenotypic changes throughout the clinical course could facilitate more efficient monitoring and support clinical decision-making. In some centers, the design of radar plots used during follow-up is largely guided by intuition or trial-and-error. The high number of channels available in modern flow cytometers greatly expands the design space, making it difficult to assess whether a given radar plot is truly suitable for a specific monitoring or analytical task, which in turn reduces its reliability (156). In recent years, dimensionality reduction techniques have increasingly emerged as powerful tools for visualizing and interpreting high-dimensional, multiparametric immunophenotypic data (157). These methods transform complex phenotypic patterns into more comprehensible and trackable representations. In the context of leukemia, ML tools such as Uniform Manifold Approximation and Projection (UMAP) and t-distributed Stochastic Neighbor Embedding (t-SNE) are particularly useful for the dynamic monitoring of LAIPs (158). By employing these dimensionality reduction techniques, clinicians are able to quickly and intuitively visualize temporal shifts in the immune landscape, thereby enabling a clearer and more effective evaluation of remission status and relapse dynamics. As dimensionality reduction tools, cloud-based software platforms such as Cytobank and OMIQ can be utilized, alongside programming environments like FlowJo (159, 160). **Figure 3** shows t-SNE-based dimensionality reduction visualizations at different time points for an MPAL case with both myeloid and T blasts identified. In bone marrow samples taken at eight different time points, myeloid blasts decrease over time, while T blasts show a more stable pattern.

## 4 Monitoring with omic technologies

In the early years, the diagnosis and classification of AML were primarily based on the French-American-British (FAB) system introduced in 1976, which relied on the morphological and cytological features of blast cells; in subsequent years, this framework was further refined by the WHO through the integration of cytogenetic abnormalities and molecular alterations (161). Despite major advances in genomic profiling, AML remains clinically challenging due to its profound molecular heterogeneity, encompassing diverse mutational patterns, epigenetic alterations, and metabolic adaptations that drive relapse and treatment resistance, thereby limiting therapeutic efficacy and prognostic precision (162). The BeatAML Consortium conducted a comprehensive study integrating genomic and transcriptomic profiling with ex vivo drug sensitivity testing in more than 800 AML samples. This consortium study extended beyond traditional genetic classifications to identify subtype-specific therapeutic vulnerabilities and resistance mechanisms, linking molecular heterogeneity to functional outcomes and establishing a multidimensional framework for AML characterization with direct relevance to precision medicine (162, 163). Accordingly, the integrated application of multi-omics approaches provides a complementary and in-depth perspective on the biological basis of disease progression, treatment response, and relapsed/refractory states, thereby reinforcing IM strategies and supporting more informed clinical decision-making. This holistic framework has the potential to mitigate relapse risk and improve long-term survival outcomes (164).

Genomic analyses elucidate the clonal evolution and mutational dynamics of leukemic cells underlying AML relapse, highlighting the genetic heterogeneity of the disease. Clonal expansions and newly acquired mutations emerging at relapse may contribute to molecular pathogenesis, while pre-existing subclonal alterations present at diagnosis can shape the etiology of relapse. Whole-genome sequencing (WGS) analyses have demonstrated that mutations in FMS-like tyrosine kinase 3 (FLT3), nucleophosmin 1 (NPM1), DNA methyltransferase 3 alpha (DNMT3A), and NADP-dependent isocitrate dehydrogenase (IDH2) identified at diagnosis are largely preserved in relapse samples. In contrast, relapse-focused investigations have revealed that FLT3-internal tandem duplication (ITD), tumor protein p53 (TP53), proto-oncogene c-KIT (KIT), runt-related transcription factor 1 (RUNX1), and Wilms tumor 1 (WT1) mutations were absent at diagnosis and were instead acquired during disease relapse, with each mutation occurring in a different patient. While gene fusion analyses indicate the maintenance of relatively stable genomic patterns throughout disease progression, cytogenomic assessments point to an increased genetic diversity in relapsed cases. Patterns of clonal evolution were found to be patient-specific, with some cases exhibiting linear evolutionary trajectories and others demonstrating branching clonal architectures. Collectively, these findings support the concept that AML relapse represents a biologically heterogeneous process shaped by diverse clonal evolution models rather than a uniform mechanism (165, 166). Mutations in AT-rich interaction domain 1A (ARID1A) and colony-stimulating factor 1 receptor (CSF1R) have been shown to be recurrently acquired genetic events during relapse in acute myeloid leukemia. In contrast, recurrent somatic mutations involving H3.3 histone A (H3F3A) and upstream binding transcription factor (UBTF) appear to represent age-specific molecular features in relapsed AML. Accordingly, H3F3A mutations are confined to adult relapse cases, whereas UBTF mutations are observed exclusively in pediatric relapsed AML (167). Taken together, these observations indicate that, as mutations present at diagnosis may be lost at relapse while new alterations emerge, temporal genomic monitoring is essential to maximize clinical benefit in relapsed AML. Such an approach supports the selection of individualized, evolution-adapted targeted therapies (168).

Transcriptomic analysis by RNA sequencing was performed on pre-treatment samples from adult AML patients younger than 60 years. All patients achieved complete remission following standard “7 + 3” induction chemotherapy. After filtering out gene expression signals associated with established prognostic mutations (CCAAT/enhancer binding protein alpha-CEBPA, nucleophosmin 1-NPM1, and FLT3-ITD), a robust 10-gene expression signature predictive of relapse was identified. This signature comprises seven protein-coding genes (growth arrest–specific 6 (GAS6), pleckstrin and Sec7 domain–containing 3 (PSD3), phospholipase C beta 4 (PLCB4), dexi homolog (DEXI), p53 cofactor (JMY), neuropilin 1 (NRP1), and chromosome 10 open reading frame 55 (C10orf55) and three long non-coding RNAs. In multivariable analyses, the 10-gene signature remained strongly associated with relapse risk after adjustment for FLT3-ITD, CEBPA, and NPM1 mutation status. Importantly, this signature demonstrated high predictive performance for relapse in an independent validation cohort from The Cancer Genome Atlas (169). scRNA-seq data have revealed marked transcriptional shifts between diagnosis and relapse, along with coordinated alterations between the tumor cells and the immune microenvironment. A relapse-enriched leukemic cell population was identified, characterized by transcriptional signatures associated with cellular quiescence, stemness, and cell adhesion. This relapse-enriched leukemic cell population indicates an enrichment of non-cycling, stem cell–like leukemic cells with enhanced adhesive properties within the bone marrow stroma. Such features are thought to confer protection against cytotoxic chemotherapy, which primarily targets cycling cells, as well as against immune-mediated responses. In addition, reduced expression of Major histocompatibility complex (MHC) class II molecules was observed in relapse-enriched leukemic cells, suggesting that immune evasion may contribute to disease relapse and represent a broader phenomenon that may not be readily captured by bulk RNA-sequencing approaches (170). In addition, ML-based gene expression network analyses have identified increased CD6 expression and decreased insulin receptor (INSR) expression as critical regulatory nodes associated with AML relapse, highlighting their potential role in disease progression and treatment resistance (171). Overall, it can be concluded that AML relapse is driven by a highly heterogeneous set of mechanisms involving complex genomic and transcriptomic interactions together with dynamic alterations in the tumor microenvironment.

Proteomic studies enable direct characterization of the molecular phenotype of AML and therefore facilitate improved identification of potentially druggable targets. Comprehensive proteomic analyses of bone marrow supernatant have revealed that clusters exhibiting similar proteomic profiles are associated with additional sex combs like 1 (ASXL1), TP53, and RUNX1 mutations, which are recognized as adverse-risk factors in the WHO classification of hematopoietic and lymphoid tissue tumors. Furthermore, testosterone levels have been found to display a close correlation with these high-risk mutations. In addition, testosterone appears to drive AML pathogenesis in conjunction with certain proteins enriched in metabolic pathways, including aldolase-fructose-bisphosphate A (ALDOA), amine oxidase copper-containing 3 (AOC3), and enolase 1 (ENO1) (172). In another study, proteomic profiling of leukemic cells isolated from patients with relapsed AML revealed marked expression changes at the time of first relapse. Relapse-associated leukemic cells exhibited significantly elevated levels of mitochondrial ribosomal proteins, including mitochondrial ribosomal protein L21 (MRPL21) and mitochondrial ribosomal protein S37 (MRPS37), as well as proteins involved in RNA metabolism such as DEAH-box helicase 37 (DHX37) and Ribonuclease P protein subunit p40 (RPP40). Similarly, increased expression was observed for ERCC3 (XBP), a helicase subunit of the TFIIH complex with a critical role in DNA repair, general transcription factor IIF subunit 1 (GTF2F1), a transcription factor and proteins regulating cyclin-dependent kinase activity. Collectively, these findings indicate enhanced mitochondrial function at relapse, particularly with respect to protein synthesis and energy production. In contrast, a marked downregulation of proteins associated with cytoskeletal organization and cell adhesion was observed in relapsed leukemic cells. These included myosin heavy and light chains (MYH14, MYL6, MYL12A), actin-binding structural components such as vinculin (VCL), and molecules involved in vesicular transport, secretion, and microenvironmental interactions, including integrin alpha-X (ITGAX), CD36 (platelet glycoprotein IV), and Glucose transporter type 3 (GLUT3) (173). Stratmann et al. identified relapse-associated protein signatures in patients with relapsed AML, demonstrating greater dysregulation of energy metabolism and RNA processing at the proteomic level in relapse cells (174).

Studies aimed at elucidating metabolic alterations in AML have demonstrated that elevated glutathione (GSH) levels are associated with poor prognosis and are markedly overexpressed in patients with chemotherapy-resistant disease. This critical increase in GSH levels has also been linked to disease relapse (175). In another study, cellular NAD^+^/NADH pools were shown to regulate oxidative phosphorylation (OXPHOS), and an expanded NAD^+^ pool was reported to be associated with disease relapse (176). In a comprehensive study using bone marrow–derived serum samples from patients diagnosed with AML, levels of alanine, valine, glycine, and glutamic acid were found to be increased in samples obtained at the time of relapse compared with controls. In relapsing patients, significantly elevated levels of palmitic acid, glyceric acid, threonic acid, propionic acid, hydroxylamine, and monomethyl phosphate were also detected relative to individuals in the control group. Collectively, these findings indicate the presence of a distinct metabolic reprogramming during AML relapse and highlight the need for more in-depth metabolomic profiling of samples from relapsed patients (177).

Patients harboring identical mutations exhibit highly heterogeneous responses to treatment. This holds true even for highly specific drugs designed to directly target the underlying oncogenic driver mutations. This observation suggests that, in addition to genomic regulation, epigenetic regulation may also play a significant role in AML pathogenesis (178). Epigenomic analyses have identified a relapse-specific chromatin accessibility signature in mutationally stable AML cases, indicating epigenetic evolution at relapse that occurs independently of additional genetic mutations. Leukemic stem cells (LSCs) exhibited markedly fewer epigenetic alterations at relapse compared with non-LSC populations, whereas epigenetic changes in non-LSC compartments reflected the overall evolutionary trajectory of the bulk leukemia. Using mitochondrial single-cell ATAC-seq (single-cell ATAC-seq combined with mitochondrial sequencing), diagnostic clones were longitudinally tracked through relapse, revealing that distinct mitochondrial clones converge toward highly similar chromatin accessibility profiles at relapse. These findings provide evidence for convergent epigenetic evolution as a defining feature of relapsed AML (179). In addition, recent studies have demonstrated that epigenetic aging is accelerated in AML compared with healthy individuals (180). Evaluating the relationship between epigenetic clock and AML relapse may be beneficial in monitoring for recurrence.

When these findings are considered together, it becomes evident that unraveling the mechanisms of relapse in AML requires moving beyond clinical follow-up strategies that rely solely on standard observational parameters, which often fall short in providing personalized insights. In relapsed AML, where OS after relapse remains disappointingly low, efforts to improve outcomes must focus on gaining a deeper understanding of the underlying molecular processes and their interplay, rather than depending on superficial metrics alone.

In the context of allo-HSCT where immune regulation is a key determinant of disease control, broadening the scope of IM is essential. This means expanding beyond routine parameters and incorporating multi-layered omics approaches, genomics, transcriptomics, proteomics, epigenomics, and metabolomics, to build robust prognostic ML models that can drive meaningful clinical improvements in survival. Alongside the well-recognized genomic, transcriptomic, and proteomic alterations in relapse, accompanying critical events such as energy metabolism reprogramming, splicing dysregulation, heightened mitochondrial activity, and shifts in microenvironmental interactions need to be assessed in an integrated manner. Such a comprehensive evaluation holds the promise of revealing the core biological dynamics of relapse and generating clinically actionable predictions. The predictive shortcomings of standard post-allo-HSCT monitoring for relapse can only be overcome through these integrative strategies, which address the diverse origins of treatment resistance and enable the development of truly personalized therapeutic approaches.

## 5 Minimal residual disease

High heterogeneity of AML and the evolution of clonal populations under selective pressure necessitate a multifaceted approach to post-transplant surveillance and intervention (181). This necessitates sophisticated clinical laboratory techniques for ongoing monitoring to detect early signs of relapse and guide personalized therapeutic adjustments (182). These advanced laboratory methods, including high-throughput sequencing and multiparameter flow cytometry, are crucial for identifying MRD and characterizing the genomic and immunophenotypic shifts that often precede overt relapse (181). Such precision monitoring allows for timely intervention strategies, including pre-emptive therapies, to mitigate disease progression and enhance patient survival (183). While prophylactic strategies such as myeloablative conditioning, prophylactic DLI, and early withdrawal of immunosuppression have shown promise in reducing relapse rates, they often come with increased treatment-related mortality (184). Consequently, genomically informed therapies are increasingly integrated into pre-transplant conditioning and as post-transplant maintenance or pre-emptive strategies, especially in cases of mixed or falling donor chimerism or persistent MRD (181).

DLI is used as a sophisticated immunomodulatory tool rather than a last choice of treatment for relapse. It is used as a prophylactic or pre-emptive tool. While pre-emptive DLI is used to treat overt relapse, prophylactic DLI (proDLI) is administered to patients who are in complete hematologic remission with complete donor chimerism and undetectable MRD, but who possess a high risk of recurrence due to advanced disease stage or unfavorable genetics. Studies indicate that proDLI is an effective strategy for preventing hematologic relapse in high-risk myeloid malignancies, especially for patients lacking targeted post-transplant maintenance options. CD3+ cell numbers in different alloHSCT are given in an additional file. Retrospective comparative data shows that early proDLI achieves higher 2-year leukemia-free survival (LFS) and a significantly lower cumulative incidence of relapse (CIR) compared to pre-emptive DLI (preDLI) triggered by MRD. For example, one study reported a 2-year LFS of 56.3% for proDLI versus 40.5% for preDLI, and a CIR of 23.4% versus 48.8%. Administering proDLI early (e.g., at a median of 51 days post-transplant) may exploit a window of maximal immune reconstitution susceptibility, allowing the recovering T-cell repertoire to target residual leukemia before it can establish immune evasion mechanisms (**Table 5**) (185, 186).

**Table 5:** CD3+ cell doses per kilogram of recipient weight recommended by EBLM for the first infusion (DLI1) using unmodified lymphocytes.

Time since alloSCT	Indication	MSD (CD3+ dose/kg)	MUD (CD3+ dose/kg)	MMUD/Haplo (CD3+ dose/kg)
3 Months	Prophylactic	0.1 x 10^6^	0.1 x 10^6^	0.1 x 10^6^
3 Months	Pre-emptive	0.1–0.5 x 10^6^	0.1 x 10^6^	0.1 x 10^6^
6 Months	Prophylactic	1.0 x 10^6^	1.0 x 10^6^	0.5 x 10^6^
6 Months	Pre-emptive	1.0–3.0 x 10^6^	1.0 x 10^6^	0.5 x 10^6^

MSD, Matched Sibling Donor; MUD, Matched Unrelated Donor; MMUD/Haplo, Mismatched Unrelated or Haploidentical Donor.

*This table is modified from (185).

A critical need exists to understand how these targeted therapies influence both GvHD potential and the immunogenicity of leukemic clones to the GvL effect (181). The ongoing development of novel agents, including targeted therapies and immunotherapies, which may enhance the GvL effect, is profoundly altering the treatment landscape for post-allo-HSCT AML relapse (187). For instance, preemptive DLI and other adoptive immunotherapies are being investigated to intensify the GvL effect, often guided by high-sensitivity chimerism monitoring to predict relapse earlier than conventional methods (188). Furthermore, sophisticated chimerism-based preemptive immunomodulation strategies, employing real-time quantitative PCR monitoring of peripheral blood and bone marrow samples, have demonstrated efficacy in assessing relapse risk and guiding early intervention (189). Growing evidence strongly supports that MRD detection via multi-parametric flow cytometry, molecular techniques, or chimerism analyses post-allo-HSCT can predict imminent relapse, warranting their inclusion in routine post-transplant follow-up to guide subsequent therapeutic interventions (184). Therefore, the meticulous monitoring of MRD before and after allo-HSCT is paramount for guiding personalized treatment strategies and improving patient outcomes in AML (190, 191). This underscores the necessity for clinical laboratories to integrate highly sensitive and specific assays to detect and quantify MRD, thereby enabling preemptive strategies and improving long-term survival in AML patients (192, 193). Consequently, the integration of NGS for MRD monitoring holds significant prognostic value, particularly in identifying relapse and predicting survival in AML patients after allo-HSCT (194). However, despite their utility, the predictive capacity of pre-transplant and early post-transplant MRD for immune-mediated eradication of leukemic cells remains a challenge, as these primarily reflect sensitivity to prior chemotherapy and conditioning regimens rather than ongoing GvL effects (195). Thus, the identification of ideal MRD markers-such as stable mutations (e.g., NPM1) or LAIP - and the optimal timing for their assessment, particularly post-transplant, remain critical areas of ongoing research (190, 196). Indeed, while multiparameter flow cytometry offers rapid turnaround times, applicability to ≥90% of AML patients, and sensitivity of at least 10^-4^, its reliance on manual analysis introduces inter-observer variability due to phenotypic heterogeneity and limits deeper immunophenotyping, necessitating standardized, automated, and computational DfN approaches (155, 197, 198). Conversely, molecular methods like quantitative/digital droplet PCR and NGS provide superior sensitivity 10^-4^−10^-6^ for trackable targets and broader detection but are applicable to only 40-60% of patients, with longer turnaround times and complex bioinformatics requirements (78, 155). MRD testing in AML represents a complex assessment process, leveraging diverse methodologies like multiparameter flow cytometry, quantitative or digital droplet PCR, and NGS to detect leukemia-specific biomarkers undetectable by routine pathological evaluation (199). These advanced techniques provide prognostic information beyond molecular risk classification at diagnosis, facilitating personalized treatment strategies-such as intensification or monitoring intervals-and improving patient outcomes (196, 197). The presence of MRD following induction or consolidation therapy, or around the time of HSCT, consistently identifies patients at high risk of disease recurrence and diminished survival, irrespective of other risk factors (200). Therefore, achieving MRD negativity prior to allo-HSCT is increasingly recognized as a critical prognostic factor influencing post-transplant outcomes, with accumulating evidence demonstrating a higher risk of relapse and poorer survival in MRD-positive patients (201). Despite these advancements, crucial questions persist regarding the optimal timing, frequency, and specific methodologies for MRD assessment, alongside the precise ways in which therapeutic interventions should be adapted based on these results (199). The inherent heterogeneity of acute myeloid leukemia, however, means that a single, universally applicable sensitive method for MRD detection remains elusive (202). Multiparameter flow cytometry, for instance, can detect MRD in up to 90% of AML patients, identifying LAIPs with a sensitivity of at least 1 in 10^-4^ (155, 197). However, the effectiveness of multiparameter flow cytometry in MRD detection is constrained by the variability of aberrant immunophenotypes and potential changes in these phenotypes during disease evolution and clonal selection (203, 204). This inherent variability can lead to inconsistent quantitation based on operator expertise, thereby limiting the utility of deeper immunophenotyping for further optimization (198). To address these limitations, the refinement of multiparameter flow cytometry protocols, such as optimizing LAIPs and establishing therapy-specific MRD cut-offs, is essential for improving accuracy and clinical utility (155). Molecular methods like NGS and quantitative PCR offer enhanced sensitivity and broader applicability to detect MRD, particularly in specific AML subsets, despite their longer turnaround times and complex bioinformatics requirements (197, 205, 206). Recent advancements in NGS allow for comprehensive surveillance of MRD at the time of response, which can predict outcomes and tailor post-remission strategies (207, 208). The 2022 ELN recommendations underscore the critical role of MRD assessment in guiding risk classification and therapeutic decisions (161). The updated recommendations from the ELN-DAVID consortium for MRD in AML emphasize the integration of various technologies, including NGS, for comprehensive MRD assessment, recognizing its importance in prognostic, predictive, monitoring, and efficacy-response evaluations (196, 209). Specifically, NGS provides broad and deep interrogation of various lesions at decreasing costs, offering a promising avenue for longitudinal, home-based testing that could overcome the limitations of single time-point assessments (152). This evolution necessitates the development of highly specific and sensitive techniques that are adaptable to the dynamic molecular landscape of AML, enabling timely and accurate relapse prediction (210). Given the complexities of clonal evolution and immunophenotype shifts, integrating multiple MRD assessment methods, such as flow cytometry and NGS, is crucial to provide a comprehensive understanding of disease status and prevent misdiagnosis (211). Furthermore, advancements in error correction technologies for NGS can significantly improve the LOD to better than 10^-7^, surpassing conventional NGS’s typical error rate of approximately 0.1% (196). This enhanced sensitivity allows for more precise monitoring of sub-clonal populations that may drive relapse, thereby improving the prognostic value of MRD assessment (212). The adoption of such advanced sequencing technologies, including ddPCR, offers a quantitative measure of actionable gene mutations and rare fusion transcripts with sensitivities comparable to or even exceeding qPCR, reaching 10–^4^ to 10^-5^ (78). Such methodological advancements are particularly critical for monitoring common PCR MRD leukemia targets like promyelocytic leukemia-retinoic acid receptor alpha (PML-RARA), core-binding factor subunit beta-myosin heavy chain 11 (CBFB-MYH11), RUNX1- RUNX1 translocation partner 1 (RUNX1T1) and NPM1 mutations, where molecular MRD relapse can precede morphological relapse, enabling pre-emptive interventions (198).

While some mutations, such as NPM1 and certain chimeric gene fusions, are highly stable and less susceptible to clonal evolution, making them ideal targets for molecular monitoring, others like FLT3 or RUNX1 are more prone to shifts (213). Consequently, a comprehensive molecular MRD panel should encompass a broad spectrum of genetic alterations to account for the polyclonal nature of AML and the potential for differential clonal responses to therapy (214). This integrated approach allows for the detection of MRD across a wider patient population, overcoming the limitations of single-gene targets that only apply to approximately 40% of AML patients (215).

It has been demonstrated that germline and somatic reduction of human leucocyte antigen (HLA) heterogeneity enhance the risk of leukemic recurrence. Preexistent germline-encoded low evolutionary divergence of class II HLA genotypes constitutes an independent factor associated with disease relapse and that acquisition of clonal somatic defects in HLA alleles may lead to escape from GvL control. However, the roles of some immune cells in the TME have not yet been identified. Further clarification of the AML pathophysiology is expected to lead to the necessity of measuring microenvironment elements and metabolic events leading to a holistic approach for MRD than exploring the cells only. In light of presently available information, there is need for more studies to determine if immune dysfunction is a prerequisite or a consequence of MRD reappearance.

### 5.1 Comparison of methods for MRD measurements

The strategic integration of multiple MRD assessment methodologies, including high-sensitivity flow cytometry and advanced molecular techniques, allows for a more robust and comprehensive evaluation of residual leukemic burden (216).

This multifaceted approach ensures that subtle changes in disease status are not missed, addressing the limitations inherent in relying solely on a single platform (217). For instance, while multiparameter flow cytometry offers broad applicability in detecting LAIPs, its diagnostic performance can be low, and its lack of harmonization across studies limits data comparability and prognostic value (155). In contrast, molecular methods, particularly those leveraging NGS, offer superior sensitivity and specificity, enabling the detection of elusive clonal populations that may escape immunophenotypic detection, if they are available (197, 218). For instance, although RNA-based fusion panels and DNA-based long-read sequencing hold promise for identifying cryptic rearrangements at diagnosis, their current sensitivity and cost-effectiveness limit their utility for routine MRD monitoring (78). Conversely, PCR-based techniques demonstrate superior sensitivity for established molecular markers, However, a negative MRD result does not definitively indicate disease eradication, as patients may still relapse even when the detected disease level is below the assay’s threshold linked to prognosis (196). An ideal MRD monitoring technique must therefore possess high sensitivity to detect residual cells at very low levels, be reproducible, rapid, and broadly applicable to a diverse patient population (206). This highlights the ongoing challenge of establishing a gold standard for MRD assessment that balances sensitivity, specificity, and accessibility across varied clinical settings and patient profiles (219). To address these challenges, advanced flow cytometry techniques are being developed, including multicolor panels that incorporate a broader range of LAIPs and DfN aberrant immunophenotypes to enhance diagnostic and monitoring capabilities (161). These advancements in flow cytometry aim to overcome the inherent limitations of conventional methods by increasing the depth of analysis, thereby improving the detection rates for MRD. Guidelines from the ELN and other international bodies provide recommendations for optimal antibody combinations, timing of assessment, and interpretation of results to standardize MRD monitoring in AML (200). ISCT guidelines for flow cytometric MRD analysis recommend an eight-color panel and a minimum of two million events to achieve the sensitivity needed for reliable detection (197, 198). Cut-off value determination is critical for clinical decision-making, with common thresholds ranging from 0.01% to 0.1% depending on the specific leukemia subtype and the time point of assessment (198, 220). Despite these advancements, persistent challenges remain in standardizing multiparameter flow cytometry across different laboratories due to variations in antibody clones, instrument settings, and data analysis pipelines, which can impact the comparability and prognostic utility of MRD results (221).

However, despite these technical refinements, flow cytometry still faces challenges related to inter-laboratory variability and the need for highly skilled personnel, underscoring the ongoing demand for standardized protocols and advanced computational analysis to enhance its clinical utility (198, 222). Molecular approaches, such as NGS and ddPCR, offer the advantage of analyzing numerous genetic alterations in a single assay, with turnaround times of less than five days and requiring less input material than multiple parallel PCR assays (223). In this context, novel approaches such as ddPCR are emerging as highly sensitive and quantitative tools for MRD monitoring, particularly for rare fusion transcripts and mutations, demonstrating comparable or superior analytical performance to traditional qPCR (78). ddPCR offers enhanced sensitivity, ranging from 10^-4^−10^-6^ and improved robustness in absolute quantification compared to qRT-PCR (78, 197, 211). This third-generation PCR technology bypasses the need for a standard curve, providing direct and absolute quantification of target DNA, which is particularly advantageous for monitoring low-abundance MRD targets (224). While ddPCR offers significant advantages in sensitivity and direct quantification for MRD detection, particularly in challenging scenarios where minimal residual leukemic cells are present, it still shares the limitation of being primarily applicable to known genetic targets identified at diagnosis (225). Additionally, the cost and specialized equipment required for ddPCR may limit its widespread adoption in all clinical laboratories, emphasizing the need for further standardization before broad clinical implementation (226). NGS offers a promising alternative by allowing for the simultaneous detection of multiple genetic aberrations, including single nucleotide variants, insertions, deletions, and copy number variations, thus providing a more comprehensive genomic landscape of residual disease (227). This multiplexing capability enables the detection of multiple clones, which is crucial for identifying emerging resistant subclones during therapy, but the high cost of NGS technology often restricts its routine clinical applicability (224). Despite the commercial availability of NGS technology since 2011, a lack of standardization in bioinformatics pipelines and interpretation protocols hinders its widespread adoption for routine MRD monitoring (196, 228). However, high-sensitivity error-corrected NGS approaches overcome some of these limitations, achieving sensitivities up to 10–^6^ for specific mutations like FLT3-ITD and NPM1, thereby enhancing their utility for MRD detection (197). Conversely, ddPCR, while offering comparable sensitivity to NGS for indels and exhibiting a faster turnaround time, necessitates allele-specific primers, complicating experimental procedures and increasing costs with a high number of parallel experiments (222). For both PCR/qPCR and NGS-based methods, DNA extracted from bone marrow, peripheral blood, or circulating cell-free DNA (cfDNA) can serve as input material for MRD detection (223). However, it is crucial to note that the choice of sample type can significantly influence the detection sensitivity, with bone marrow typically offering higher cellularity and thus better representation of residual leukemic cells compared to peripheral blood, especially for mutations not commonly found in circulating cfDNA (196). Despite these advancements, the inherent reliance on *a priori* knowledge of patient-specific mutations limits their utility in cases where the clonal architecture evolves or new mutations emerge at relapse (197). NGS offers a more comprehensive approach to address this challenge by simultaneously screening for a broader spectrum of mutations, yet its standardization for routine diagnostics and harmonization across laboratories remains a significant hurdle (196, 229). Furthermore, while NGS can detect a broad spectrum of targets for MRD monitoring, it is generally less sensitive than qPCR for specific targets (78). This sensitivity gap can lead to situations where NGS fails to detect mutations such as FLT3-ITD and NPM1 that are reliably identified by more sensitive qPCR-based methods, underscoring the complementary roles of different platforms in comprehensive MRD assessment (223). Nevertheless, specialized NGS assays have been developed to enhance sensitivity for specific mutations, such as FLT3-ITD, demonstrating superior performance over conventional methods in MRD monitoring and prognostic correlation in AML patients post-HSCT (230). This capability is particularly vital given the genotypic and phenotypic drifts that can occur during treatment, which NGS can reliably detect by assessing patient-specific mutations at diagnosis and throughout the disease course (193).

Currently, MRD analysis relies on invasive, serial bone marrow (BM) biopsies, which complicate sample availability, processing time and impact the patient experience. Sometimes finding a positive result can generate more questions than answers, causing anxiety for both the patient and the physician. Peripheral blood (PB) evaluation has shown promise in detecting MRD and is now recommended by the European Leukemia Net for AML for certain genetic abnormalities as mentioned above. Even though PB-based sampling allows for more frequent testing intervals and better temporal resolution of malignant expansion while sparing patients additional invasive procedures, the reliability needs to be shown by further studies in order to be accepted in clinical routine work up (231).

Chimerism analysis, which tracks donor and recipient cell populations post-transplantation, serves as another critical tool for relapse monitoring, particularly when integrated with molecular MRD techniques for a more comprehensive assessment (206). While the measurement of mutational stability remains a challenge due to factors like mutational shifts and multiclonality, NGS offers enhanced sensitivity to overcome these limitations for MRD monitoring (232). However, the inherent sensitivity of NGS is often constrained by its background error rate, and a consensus on methodologies to mitigate these errors is still in development (203). Use of flow cytometry and NGS together with other clinical laboratory techniques can overcome individual limitations and provide a more robust assessment of relapse risk in post-transplant AML patients, especially when considering the dynamic nature of chimerism and MRD (233). Molecular MRD detection by NGS has been shown to correlate with AML relapse and survival, although challenges persist in distinguishing AML-specific mutations from clonal hematopoiesis (234). However, studies utilizing high-sensitive NGS have indicated that combining MRD methods may not always yield additional prognostic benefits beyond FLT3-ITD and NPM1 NGS MRD, particularly when considering specific mutations (197). Despite the prognostic significance of certain mutations like FLT3-ITD, the stability of these mutations over time can be a concern, as some may become undetectable or new subclones with different mutational profiles may emerge at relapse, complicating long-term MRD monitoring (219). For instance, persistent IDH2 mutations detected before allo-HSCT are associated with worse outcomes, even in the absence of co-mutated NPM1 and/or FLT3-ITD (197). This highlights the importance of comprehensive molecular profiling beyond commonly tracked mutations to effectively predict and monitor relapse risk post-transplantation (235). Furthermore, serial monitoring with NGS-MRD can effectively detect the reappearance of mutant clones prior to overt relapse, providing an early warning system for therapeutic intervention (194). Recently published articles by Shaffer and Wienecke continues to emphasize the prognostic significance of NGS-based MRD detection in AML patients post-allo-HCT, with a focus on improving the sensitivity and specificity of these assays for earlier detection of impending relapse (199, 206). The clinical utility of these advanced NGS approaches is further enhanced by their ability to provide longer lead times to relapse, particularly for mutations in epigenetic modifier genes and stable mutations known from diagnosis (206).

New techniques such as error-corrected NGS (ecNGS) significantly improve sensitivity, enabling the detection of molecular relapse several months before clinical manifestation, even in cases of low-level MRD (206). This enhanced sensitivity is crucial given that molecular MRD detection by NGS is associated with significantly higher relapse rates (236). However, the inherent instability of certain mutations, such as those in signaling genes like NRAS and FLT3, presents a challenge for long-term MRD monitoring, as these mutations can be gained or lost between diagnosis and relapse. In contrast, mutations in genes such as NPM1 and IDH1/2 are generally more stable and thus serve as more reliable markers for long-term MRD assessment (217). Moreover, the selection of appropriate MRD markers is paramount, as mutations associated with clonal hematopoiesis of indeterminate potential or myeloid differentiation genes may persist in remission and not signify actual leukemic relapse, necessitating careful interpretation of NGS results (196). Therefore, identifying a suitable patient-specific mutation panel for NGS-MRD monitoring, prioritizing stability and specificity, is critical for accurate relapse prediction and guiding post-transplant interventions.

Despite advancements, several challenges persist in effectively monitoring AML relapse following HSCT, predominantly stemming from the complex biology of the disease and the limitations of current diagnostic techniques. One such challenge involves the identification of optimal monitoring intervals, as studies indicate that frequent sampling is necessary to capture the molecular kinetics of relapse, with a substantial proportion of patients becoming MRD positive within months of overt relapse (206). For instance, monthly peripheral blood NGS-MRD monitoring has been shown to detect 64% of relapses, an improvement over quarterly monitoring which detects 38% (206). However, the feasibility and cost-effectiveness of such intensive monitoring schedules necessitate further investigation to balance clinical benefit with practical implementation. Furthermore, while error-corrected NGS MRD assays offer high sensitivity and the potential to monitor a wide range of target mutations, their current high cost and limited availability outside of academic studies present significant barriers to widespread adoption (203). This underscores the critical need for further development and validation of more accessible and affordable NGS-MRD technologies that can be routinely implemented in clinical laboratories (223, 237). Another complexity arises in differentiating donor-derived mutations from those originating from disease relapse, particularly given the dynamic nature of chimerism post-transplant (238). The presence of donor-derived clonal hematopoiesis, indistinguishable from recipient leukemia by conventional methods, further complicates MRD interpretation, particularly when employing highly sensitive techniques like NGS (193). Accurate discrimination between recipient-derived leukemic cells and donor-derived hematopoietic stem cell mutations is essential for proper relapse detection and guiding subsequent therapeutic strategies (206). Consequently, advanced computational algorithms and a thorough understanding of patient-specific genetic landscapes are becoming increasingly vital for distinguishing between these complex scenarios and ensuring timely and appropriate clinical interventions (239). Moreover, the intricate nature of data analysis and interpretation poses further obstacles, demanding specialized expertise and resources for accurate assessment of clonal populations (240).

Future methods of MRD detection are moving towards integrating multi-omic approaches that combine genomics, transcriptomics, and proteomics to provide a more comprehensive view of residual leukemic burden and identify novel therapeutic targets. One promising avenue involves the use of circulating cfDNA to monitor MRD, offering a non-invasive alternative to bone marrow biopsies (223). This approach holds significant potential, particularly for frequent monitoring, though standardization of collection and analysis methods remains a critical step for clinical implementation (239). Disadvantage of cfDNA analysis include its short half-life and the challenges in distinguishing disease-related mutations from those associated with clonal hematopoiesis (223). Furthermore, the detection of leukemia stem cells through methods like flow cytometry, particularly focusing on CD34^+^CD38^neg^ phenotypes in conjunction with aberrant markers, is emerging as a critical prognostic indicator, having been validated in prospective trials despite challenges with CD34^neg^ phenotypes (197). However, the subjectivity and time-intensive nature of manual gating in flow cytometry necessitate the exploration of automated analytical solutions to enhance standardization and efficiency (196). Moreover, advancements in single-cell sequencing technologies are poised to revolutionize MRD assessment by enabling the identification of rare leukemic subclones and providing insights into their genomic evolution, offering a more granular understanding of residual disease heterogeneity (241). These advanced techniques, such as next-generation flow cytometry and ddPCR, offer enhanced sensitivity and specificity compared to traditional methods, allowing for more precise detection of MRD and better prediction of relapse risk (224). For instance, next-generation flow cytometry provides superior sensitivity by allowing the analysis of millions of cells, thereby improving the detection of rare leukemic populations below the limits of conventional flow cytometry (198). Similarly, digital PCR offers a more precise quantification of molecular targets, enabling the detection of MRD at concentrations far below the detection limits of traditional quantitative PCR assays (155). Despite these technological advancements, the optimal integration of these diverse MRD assessment modalities into a unified, clinically actionable framework remains a significant challenge, requiring robust validation studies and standardized protocols (155, 242).

Surface Enhanced Raman Scattering (SERS) is label free method which identifies malign cells by their intrinsic molecular signatures and vibrational fingerprints rather than their ability to bind antibodies (243). This is a superior feature in comparison to immunophenotyping, especially when the sample quality is poor or cell viability is compromised. The analytical sensitivity and clinical utility of established and emerging MRD methodologies are summarized in **Table 6**.

**Table 6:** Analytical performance and clinical applicability profiles of techniques used for MRD monitoring.

Method	Sensitivity (LOD)	Applicability	Key strengths	Main limitations
FISH	10^-2^-10^-3^	~50% of cases	Fast; captures interphase cells.	Lowest sensitivity; requires pre-existing abnormal karyotype.
MFC (Multiparametric Flow)	10^-3^-10^-5^	100% of cases	Rapid turnaround (hours); detects viable cells.	Standardization difficulties; requires fresh samples; immunophenotypic shifts.
Next-Gen. Flow (NGF)	10^-5^-10^-6^	>95% of cases	Highly standardized (EuroFlow); deeper sensitivity than MFC.	Requires specialized expertise and high cell counts.
Real-time qPCR	10^-4^-10^-6^	40–50% of cases	Highly sensitive; standardized for known markers (*NPM1*, fusions).	Restricted to specific genetic markers; RNA instability in RT-qPCR
ddPCR (Digital PCR)	10^-4^-10^-5^	Marker-dependent	Absolute quantification; easier to standardize than qPCR	Higher cost than standard qPCR
NGS	10^-4^-10^-7^	>95% of cases	Tracks multiple mutations; detects clonal evolution; personalize	High cost; slow (days); confounded by CHIP or germline mutations.
SERS (Surface Enhanced Raman Scattering)	10^-5^-10^-7^	Emerging	Label-free; ultra-fast (<1 min); does not require cell viability	Currently proof-of-concept; limited clinical validation.

The table contrasts traditional techniques (FISH, MFC) with high-sensitivity next-generation technologies (NGF, NGS, PCR variants) and emerging label-free approaches (SERS) regarding their sensitivities (LOD), advantages, and operational limitations.

LOD, Limit of Detection; FISH, Fluorescence in situ Hybridization; MFC, Multiparameter Flow Cytometry; NGF, Next-Generation Flow; qPCR, Quantitative Polymerase Chain Reaction; ddPCR, Droplet Digital PCR; NGS, Next-Generation Sequencing; SERS, Surface-Enhanced Raman Scattering; CHIP, Clonal Hematopoiesis of Indeterminate Potential; NPM1, Nucleophosmin 1.

Timeline for MRD assessment should be carefully considered, as the timing of monitoring impacts its clinical utility (198). For instance, post-transplant MRD assessment at day 30, day 100, and then every three months for two years is a commonly accepted schedule, but variations exist depending on the transplant center and the patient’s risk profile (197). However, more frequent monitoring, especially within the first six months post-transplant, may be warranted for high-risk patients or those with persistent MRD pre-transplant (196). The predictive accuracy of multiparameter flow cytometry in detecting MRD is significantly influenced by the specificity and sensitivity of the LAIPs utilized, necessitating refined methodologies and therapy-specific cut-offs for optimal clinical utility (155). The advent of sophisticated bioinformatics tools further enables the integration of multi-modal data, allowing for a more holistic interpretation of a patient’s MRD status and providing deeper insights into disease biology. For example, computational approaches like FlowSOM are gaining traction for automated analysis of multiparametric flow cytometry-MRD diagnostics, promising to reduce interobserver variability and harmonize results across laboratories (244). Furthermore, efforts are underway to establish universal standards and guidelines for MRD testing, ensuring consistent and comparable results across different institutions and enhancing the reliability of MRD-guided treatment decisions (197, 224). Despite these advancements, a key challenge lies in the current standardization of MRD detection, with significant differences in prognostic value observed, particularly among smaller centers, highlighting the urgent need for harmonized approaches (79, 197). Standardization efforts are particularly critical for molecular MRD testing, where factors such as gene targets, tissue types, and bioinformatics pipelines all contribute to variability in prognostic and predictive relevance (196). This variability in cutoffs, coupled with the heterogeneity in assay design and analysis strategies, poses substantial challenges for cross-trial comparisons and the development of universally applicable guidelines (197, 209). The inter-laboratory variabilities as reported in CIBMTR analysis are challenging for clinical decision making (245). The pre-MEASURE study is aiming to minimize the inter-laboratory variabilities as do other ongoing studies of ELN and International Society for Cell and Gene Therapy – ISCT (199). Qualification and through validation of each MRD method in a laboratory working under strict quality control criteria is critical for clinical decisions. The ELN-DAVID group, in collaboration with UK-NEQAS, is currently conducting inter-laboratory tests on MFC (including LSC), qPCR, and UHS-NGS MRD and declare that multicenter collaboration is encouraged. These comparative studies should assess the turnaround time, cost, sensitivity, and clonal evolution effects across methods (209).

Consequently, the harmonization of methodologies, including the establishment of standardized panels for multiparameter flow cytometry and consensus on molecular targets for NGS, is paramount to ensure the reliable interpretation and clinical utility of MRD assessments (196, 199). The integration of these standardized approaches is essential for leveraging MRD as a robust biomarker for guiding pre-emptive interventions and personalized treatment strategies in post-transplant AML (202, 246). Such strategies would involve, for instance, tailoring post-transplant immunosuppression or initiating targeted therapies based on MRD kinetics and the identified molecular aberrations of residual leukemic clones, thereby optimizing patient outcomes (197, 208, 221). The continuous evolution of these methods, encompassing both sensitivity enhancements and broader applicability, highlights the need for ongoing validation and refinement to ensure their clinical relevance in detecting and monitoring MRD (155, 196, 199, 247). This necessitates rigorous validation studies to establish the predictive power of evolving MRD assays for guiding therapeutic decisions and improving patient outcomes in the context of post-HSC transplant AML (196, 197, 211). The utility of MRD assessment extends beyond prognosis, increasingly guiding clinical decision-making, particularly in the context of post-HSC transplantation (196, 248).

To conclude, the robust and standardized integration of clinical laboratory techniques for MRD detection is essential for predicting relapse, informing therapeutic adjustments, and ultimately improving survival in AML patients after HSCT (194, 201, 207, 249).

## 6 Data integration and reporting in clinical laboratories

Following allo−HSCT, IM in patients with AML generates high−dimensional, longitudinal datasets that capture cellular composition, functional immune states, and dynamic changes over time. While individual immune parameters may provide limited insight in isolation, their clinical relevance emerges primarily through systematic integration and structured interpretation within the clinical laboratory setting. In this context, the primary challenge is no longer the generation of immune data, but rather the transformation of heterogeneous measurements into reproducible, interpretable, and clinically actionable laboratory reports that can inform relapse risk assessment and guide interventions such as adjustment of immunosuppression, pre−emptive therapy, or intensified MRD surveillance (250).

Clinical laboratories occupy a pivotal position in this process, serving as the interface between complex analytical outputs and bedside decision−making. Effective data integration requires not only technical harmonization of multiparametric immune measurements but also standardized analytical frameworks that preserve biological meaning while minimizing observer variability and inter−center variability (251). Moreover, reporting strategies must balance analytical sophistication with transparency, ensuring that clinicians receive concise, reliable summaries rather than unfiltered or opaque computational outputs (252). Consequently, data integration and reporting should be viewed as a continuum, in which preprocessing, analytical modeling, and intelligent presentation collectively determine the clinical utility of IM after transplantation (253). Building on these principles, the following sections outline key approaches to standardized data acquisition, multiparametric integration, and risk−oriented reporting, with particular emphasis on methodologies that are feasible within routine clinical laboratories and capable of supporting longitudinal relapse surveillance in allo−HSCT recipients.

### 6.1 Standardized data acquisition and preprocessing

Standardized data acquisition and preprocessing represent the indispensable foundation for any form of integrated or intelligent IM in clinical laboratories. In the post allo-HSCT setting, where immune parameters are assessed longitudinally to inform relapse surveillance in AML, technical variability introduced during sample handling, data acquisition, or preprocessing can readily obscure biologically meaningful signals. This risk is further amplified in multicenter contexts, in which even modest differences in laboratory practice may compromise cross-site comparability and erode clinician confidence in IM-based reports (254).

Evidence from multicenter IM initiatives demonstrates that harmonized acquisition strategies substantially reduce inter-laboratory variability and enable robust longitudinal analyses. Key elements include the use of validated, disease-relevant immune phenotyping panels with fixed clone and fluorochrome configurations, pre-formatted antibody cocktails, and shared panel layouts. When combined with standardized instrument setup anchored by bead-based calibration, common voltage templates, and systematic daily quality control, such approaches ensure that interindividual biological variation exceeds inter-site technical noise for most major immune subsets. These considerations are particularly critical in the allo-HSCT setting, where immune phenotypes differ markedly from healthy reference populations and are shaped by graft source, conditioning regimens, infections, and post-transplant immune modulation (255, 256). Pre-analytical and analytical standard operating procedures constitute an additional pillar of standardization. Harmonized protocols governing sample type, collection timing, storage conditions, cryopreservation, and staining workflows are essential to preserve cell viability, marker stability, and quantitative comparability over time (257, 258). Automated or semi-automated sample preparation steps may further reduce operator-dependent variability, particularly in high-throughput clinical laboratory environments supporting long-term follow-up of transplant recipients (259). Preprocessing and data analysis represent a major downstream source of variability, with manual gating remaining highly dependent on operator expertise and local conventions. Validated automated or semi-automated preprocessing pipelines have demonstrated high concordance with expert manual analysis for many commonly assessed immune subsets, while offering substantial gains in reproducibility and efficiency (260). Nevertheless, challenges persist for low-frequency or poorly resolved populations and for markers sensitive to cryopreservation or staining conditions. Accordingly, automated approaches should be implemented within structured quality control frameworks that incorporate drift detection, reference datasets, and human-in-the-loop review, rather than being treated as fully autonomous, black-box solution (261).

The importance of standardized acquisition and preprocessing becomes particularly evident when considering downstream multiparametric integration and risk-oriented reporting. The meaningful combination of immune subset distributions, functional markers, chimerism data, MRD assessments, and clinical covariates presupposes that each measurement is analytically comparable across patients and over time. Without rigorous standardization at the acquisition and preprocessing stages, apparent changes in immune risk markers may reflect technical drift rather than true biological dynamics, undermining the validity of longitudinal relapse risk assessment. Collectively, these measures should be regarded not as incremental technical optimizations, but as fundamental prerequisites for credible data integration and for the responsible deployment of automated and AI-assisted analytical frameworks in routine clinical laboratories (262, 263).

### 6.2 Multiparametric data integration and feature engineering

Multiparametric data integration and feature engineering are central to transforming heterogeneous post-transplant measurements into clinically actionable risk information. Following allo-HSCT, IM generates multiple complementary data layers, including quantitative and phenotypic recovery of lymphoid and myeloid subsets, functional immune readouts, and the longitudinal dynamics of IR (35, 258). When integrated with non-immune markers such as MRD, donor chimerism, and soluble biomarkers, these data streams provide a more comprehensive representation of the balance between immune recovery, GvL activity, and treatment-related toxicity than any single parameter considered in isolation (13, 20). Within this integrative framework, feature engineering plays a critical role in structuring complex data for downstream analysis and reporting (264). Feature extraction involves transforming raw measurements into biologically meaningful descriptors, such as ratios between immune subsets, composite indices reflecting immune competence or dysfunction, and trajectory-based metrics that summarize the tempo, stability, or disruption of immune recovery over time (256, 258). These derived features enable longitudinal patterns and functional relationships to be captured in a form that is more amenable to interpretation and clinical use (263). Feature selection subsequently refines this expanded feature space by identifying a parsimonious subset of informative variables, typically using multivariable or model-based approaches. The primary objectives are to reduce redundancy, enhance analytical robustness, and preserve interpretability, all of which are essential in the clinical laboratory context (262). Importantly, feature selection strategies must balance statistical performance with biological plausibility and reporting feasibility, as overly complex or unstable feature sets may undermine clinical confidence despite favorable predictive metrics (255). Evidence from allo-HSCT cohorts indicates that carefully engineered features-ranging from immune cell recovery dynamics to integrated MRD–chimerism profiles-can improve the prediction of relapse, NRM, infectious complications, and poor graft function. As such, multiparametric data integration and feature engineering form a critical bridge between standardized data acquisition and transparent risk stratification, enabling IM outputs that are both analytically robust and suitable for routine reporting in clinical laboratories (13).

### 6.3 Rule-based and knowledge-driven decision systems

Rule-based and knowledge-driven decision systems remain central to IM after allo-HSCT because they translate complex immunological and clinical information into clear, clinically actionable categories. Cut-off–based risk definitions are among the most widely used approaches for post-transplant biomarkers and immune parameters. Representative examples include fixed thresholds for soluble markers such as suppression of tumorigenicity 2 (ST2) or tumor necrosis factor receptor 1 (TNFR1) to stratify NRM risk, as well as DSA mean fluorescence intensity cut-offs used to assess donor suitability (264–266). Similarly, guideline-backed milestones for immune recovery, such as CD4^+^ T-cell counts exceeding 50 cells/µL by day 100, have been proposed as pragmatic indicators of adequate IR and are readily incorporated into routine laboratory reporting (267).

Consensus guideline–driven algorithms extend these single-parameter thresholds into structured, multi-step decision pathways. International and national transplant recommendations integrate disease-related risk scores, such as revised international prognostic scoring system (IPSS-R) or molecular international prognostic scoring system (IPSS-M), comorbidity indices including the hematopoietic cell transplantation specific comorbidity index (HCT-CI), and transplant-specific factors into harmonized frameworks guiding decisions on transplant indication, timing, donor selection, conditioning intensity, and post-transplant management (268, 269). From a clinical laboratory perspective, such algorithms promote standardized interpretation and reporting by embedding laboratory measurements within agreed clinical contexts, thereby facilitating comparability across centers and studies (270).

Expert-defined scoring systems further synthesize multidimensional information into composite indices that summarize overall risk or immune status as a single, interpretable value. In the allo-HSCT setting, this includes established transplant-specific scores such as the European Society for Blood and Marrow Transplantation (EBMT) score and the disease risk index, as well as more recent immune-focused tools like the Composite Immune Risk Score, which combines selected leukocyte subsets into a simple formula to predict early mortality and inform infection-prevention strategies (271, 272). These scores exemplify how domain expertise can be leveraged to balance biological relevance with reporting simplicity, while remaining accessible to clinicians. Collectively, rule-based and knowledge-driven approaches support harmonized reporting of chimerism, MRD, and immune recovery across centers and provide a stable, transparent foundation for clinical decision-making. Importantly, they also serve as a reference framework against which newer statistical and machine learning–based models are increasingly evaluated, and with which they are often combined in hybrid decision-support systems aimed at refining risk stratification without sacrificing interpretability (273).

### 6.4 Statistical and predictive modeling approaches

Statistical and predictive models bridge the gap between simple rule-based systems and complex AI frameworks, offering transparent and interpretable risk estimates essential for clinical laboratory use (274). Within the allo-HSCT setting, two model families predominate: Cox proportional hazards models are the standard for evaluating outcomes like OS and infection risk due to their ability to incorporate time-dependent covariates (275), while Fine-Gray models are specifically suited for competing risks, such as distinguishing between disease relapse and NRM (276). Because IR is inherently dynamic, static baseline analyses often fail to capture clinically relevant shifts (277). Longitudinal modeling strategies-including time-varying covariates, landmark analyses, and multi-state models-address this by linking the evolving trajectories of lymphocyte recovery and viral reactivation to future risks (278). These frameworks translate complex temporal relationships into intuitive risk trajectories or state-transition diagrams, providing the visual clarity needed for multidisciplinary decision-making (279). Furthermore, regression-derived composite risk scores allow laboratories to synthesize multiple immune and clinical variables into a single, calibrated metric. Recent methodological advances, such as penalized models and multiple-imputation strategies for missing data, ensure these techniques remain robust in real-world clinical datasets (280). Ultimately, these explainable statistical approaches provide a rigorous methodological scaffold that serves as a benchmark for evaluating and integrating more complex machine-learning architectures (281).

### 6.5 AI-based analytical frameworks

AI–based analytical frameworks have demonstrated substantial potential for improving relapse prediction after allo-HSCT in AML. By leveraging high-dimensional immune phenotyping, chimerism, and clinical data, these approaches can outperform traditional models in discrimination and pattern recognition. Nevertheless, significant methodological, operational, and regulatory barriers continue to limit their routine implementation in clinical laboratories (282). Supervised ML methods-including random forests, gradient boosting (e.g., XGBoost), support vector machines, and alternating decision trees (ADTree)-have been most frequently applied to predict post-HSCT relapse and NRM from multiparametric immune and clinical data (283). Several studies illustrate the promise of these approaches. Early post-transplant immune phenotypes analyzed by XGBoost models combined with synthetic minority oversampling (ADASYN) have achieved approximately 90% accuracy in predicting AML relapse within 30 days using high-dimensional flow cytometry data (284). Random forest models integrating chimerism and clinical variables have predicted pediatric leukemic relapse with accuracies of approximately 80–85%, while simultaneously providing feature-importance and partial-dependence plots that highlight clinically plausible drivers such as age and CD34^+^/CD3^+^ chimerism levels (285). ADTree-based models have reported relapse prediction accuracies in the range of 70–78% and offer visual decision paths that are readily inspectable by clinicians, enhancing interpretability (286).

Unsupervised and semi-supervised approaches-including t-SNE, UMAP, PhenoGraph, self-organizing maps, and FlowSOM-play a complementary role by enabling immune pattern discovery and identification of cluster-level phenotypes associated with relapse risk. However, these methods do not directly yield labeled risk estimates and typically require an additional supervised layer for clinical decision support. Despite encouraging internal performance, model robustness remains a critical concern. Many studies emphasize the need for large, heterogeneous cohorts and external validation, noting that center-specific protocols, temporal drift, and cohort composition can substantially degrade generalizability when models are transferred across laboratories (287–289).

Deep learning (DL) approaches extend ML capabilities by learning compact representations directly from high-dimensional data. Convolutional neural networks, attention-based multiple-instance learning (MIL), and transformer architectures have been applied to raw flow cytometry events, single-cell data, and multimodal inputs (290). Attention-based MIL models trained on large AML flow cytometry datasets have achieved AUROC values approaching 0.96 for AML classification and have demonstrated the ability to predict cytogenetic abnormalities while highlighting cell populations driving model decisions. In related hematologic settings, deep models and radiomics-based CNNs report accuracies ranging from approximately 70% to over 90% for disease detection or relapse prediction (291).

Theoretical advantages of DL include the ability to capture nonlinear, high-dimensional immune patterns, jointly model immune, MRD, and genomic data, and potentially standardize gating and feature extraction across centers. However, these benefits are offset by substantial limitations in the clinical laboratory context. DL models require very large, well-curated, and standardized datasets-conditions rarely met in allo-HSCT IM, where protocols and instruments vary between centers. They are also vulnerable to data drift and may fail on rare or atypical immune phenotypes without continuous monitoring. In addition, the computational, validation, and regulatory burden associated with DL (including infrastructure, MLOps, quality assurance, and explainability requirements) remains a major obstacle to accreditation and clinician acceptance (292–294).

Hybrid and ensemble models represent a pragmatic strategy to balance predictive performance with clinical interpretability. These systems combine rule-based logic with ML models using architectures such as rule-guided preprocessing, ML-assisted rule refinement, or parallel ensembles in which rule engines and ML predictors operate simultaneously and are fused. Parallel, confidence-weighted ensembles have been shown to outperform either rules or ML alone while allowing clinicians to default to guideline-based decisions when model predictions conflict with established practice (295, 296). In AML relapse monitoring after allo-HSCT, hybrid frameworks can enforce consensus MRD and chimerism thresholds as safety nets, while ML components refine individualized risk estimates within those constraints (297). Crucially, hybrid models facilitate layered explainability by combining rule-level logic, feature-importance metrics, and case-level explanation tools such as LIME, SHAP, or attention maps. This alignment with clinical reasoning makes hybrid approaches particularly attractive for laboratory reporting and multidisciplinary decision-making (298).

Within this context of increasing analytical complexity, the translation of complex AI frameworks into routine clinical laboratory practice remains constrained by substantial validation and regulatory challenges. In immune monitoring after allo-HSCT, where models are applied to high-dimensional, longitudinal, and highly heterogeneous data, rigorous technical validation is required, including internal cross-validation, external multi-center testing, robustness to dataset shift, and systematic bias analysis. Beyond analytical performance metrics such as AUC, meaningful clinical validation must demonstrate added value over established parameters (e.g., MRD and chimerism), as well as measurable effects on laboratory workflows, diagnostic accuracy, quality of care, and patient outcomes in prospective studies or randomized clinical trials (299, 300).

Current regulatory pathways for software as a medical device (SaMD) continue to face difficulties in accommodating adaptive or continuously learning algorithms, particularly in dynamic clinical settings such as post-transplant immune recovery. Limitations in task definition, phase-based evaluation, and site-specific performance assessment hamper trust, regulatory approval, and broad adoption. As a result, emerging guidance increasingly advocates multi-phase evaluation frameworks analogous to drug development, strengthened post-market surveillance, and independent third-party assessment to monitor performance drift and ensure safety across installations (301). In parallel, horizontal regulations, including the EU Artificial Intelligence Act (302), together with domain-specific initiatives such as FUTURE-AI, emphasize fairness, robustness, explainability, data quality, and traceability as core prerequisites for the deployment and sustained oversight of high-risk clinical AI systems (303).

Without systematically addressing these validation and regulatory barriers, particularly those related to data governance, algorithmic bias, transparency, and site-level monitoring—the translation of complex AI frameworks from research prototypes into routine clinical laboratory use is likely to remain slow and fragmented. Accordingly, in their current form, such systems are most realistically positioned as clinical decision-support tools that augment expert laboratory and clinical interpretation rather than as autonomous decision-making entities (304).

## 7 Future perspectives

While multiple emerging technologies hold promise for advancing immune monitoring after allo-HSCT, their clinical readiness, feasibility, and cost-effectiveness vary substantially. Accordingly, this section differentiates approaches that are approaching routine clinical implementation from those that remain confined to specialized centers or research settings, as well as from conceptual frameworks that require further validation before clinical adoption. Although recent technological advances have substantially expanded analytical capabilities, their successful translation into routine immune monitoring remains dependent on standardization, practical workflow integration, and cost-effectiveness. These considerations are equally relevant for immunological monitoring across hematological malignancies, particularly during active treatment and in the post-allo-HSCT setting.

In recent years, the number of measurable parameters has increased with the development of spectral flow cytometers. Unlike traditional flow cytometers, spectral flow cytometers can measure multiple parameters simultaneously without being limited by the number of channels and can provide immune tracking with >40 color panels. However, their integration into clinical practice is a challenging process because repeatability and the creation of easy analysis sets are more difficult (305). The ability to detect a large number of parameters simultaneously with a single panel enables comprehensive evaluation of the maturation, differentiation, and abnormalities of multiple cell lineages in immune monitoring (306). The ability to detect a large number of parameters simultaneously with a single panel enables comprehensive evaluation of the maturation, differentiation, and abnormalities of multiple cell lineages in immune monitoring. Mass cytometry, also known as Cytometry by Time-of-Flight (CyTOF), enables the simultaneous characterization of over 40 parameters per cell, allowing for the precise identification of lineage sub-populations and the detailed mapping of the tumor microenvironment. By facilitating the concurrent monitoring of intracellular signaling across multiple pathways (307), CyTOF holds significant potential for the comprehensive assessment of relapse risk and prognostic stratification following allo-HSCT. In AML, spatial monitoring is essential as it moves beyond cell counts to map leukemic blasts within their protective bone marrow niches. By uncovering single-cell interactions and microenvironmental crosstalk, this approach deciphers immune evasion and treatment resistance mechanisms that standard flow cytometry cannot detect (308). In this context, emerging spatial approaches enable integrated analyses with bone marrow structure, providing spatially resolved insights into leukemic stem cell persistence and niche-dependent immune escape mechanisms. Therefore, investigating the microenvironmental interactions between leukemic blasts and immune cells through spatial biology in post-allo-HSCT relapse cases has the potential to contribute significantly to the elucidation of relapse mechanisms. Despite their high analytical depth, these technologies remain constrained by cost, infrastructure requirements, and limited standardization, restricting their use to specialized centers.

Cell sorting has established itself as a foundational technology by enabling the isolation of pure cell subpopulations for immunological research, and it remains indispensable today by providing high-quality samples for advanced genomic and proteomic analyses (309). The high-quality, simultaneous isolation of blasts and associated immune cells from routine clinical samples elucidates the complex interactions within the microenvironment through multi-omic investigations. In the coming years, the integration of cell sorting capabilities into routine flow cytometry will facilitate the transition from analyzing bulk heterogeneous samples to evaluating highly homogeneous cell populations. This shift will not only provide profound insights for clinical research but also enable the adoption of more precise, personalized medicine approaches.

Assessment of thymic output following allo-HSCT is utilized to evaluate the capacity for *de novo* T-cell production from donor-derived progenitors and to monitor the robustness of IR. This analysis serves as a vital prognostic marker for gauging the patient’s susceptibility to opportunistic infections and understanding the dynamics between thymic recovery and GvHD (39). Integrating thymic function analysis into routine post-allo-HSCT monitoring enables the assessment of donor-derived *de novo* T-cell production, thereby facilitating the prediction of IR kinetics and defensive efficacy against infections. Although a standardized consensus by authorities for the prognostic and predictive monitoring of the allo-HSCT process is currently lacking, incorporating such parameters into routine immunomonitoring has the potential to yield positive impacts on overall survival OS. TCR repertoire analysis provides valuable insight into post-transplant immune diversity and clonal dynamics; however, its routine clinical applicability remains limited. Current TCR sequencing approaches typically require several days to weeks for data generation and interpretation and depend on specialized bioinformatic pipelines and expert-driven analysis, which restrict their use in time-sensitive clinical decision-making. In addition, comprehensive assessment of the γδ T-cell compartment necessitates dedicated panels covering Vδ1, Vδ2, and Vδ3 segments to accurately capture the human TCR-δ repertoire, a requirement that further increases technical complexity and limits standardization. Consequently, TCR repertoire analysis currently serves primarily as an advanced research tool rather than a broadly actionable clinical assay. Deep TCR repertoire profiling in this manner is expected to enable a more comprehensive evaluation of critical factors such as clonal evolution, immune escape mechanisms, and impaired GvL activity in AML patients who relapse after allo-HSCT, ultimately contributing to the development of personalized preventive and therapeutic strategies in the future.

Monitoring IR alongside remission and relapse assessments in samples collected at all routine post-allo-HSCT follow-up time points will facilitate a deeper understanding of the cellular and molecular regulation occurring throughout the pre-relapse phase. Following the integration of multi-cohort data into international databases, the comprehensive networks revealed through bioinformatics will enable us to identify the specific stages of immune reconstitution where intrinsic or extrinsic mechanisms trigger the relapse process, ultimately allowing for the elucidation of the underlying pathology. These approaches are increasingly feasible in advanced clinical laboratories and represent the most immediate opportunities for broader clinical integration.

Although highly promising, AI-based risk modeling and digital twin frameworks currently remain research-driven tools, with major challenges related to data harmonization, regulatory validation, and real-world clinical deployment. The future development of clinical AI algorithms capable of quantifying relapse risk through immune system dynamics is essential for ML-based clinical monitoring and evaluation. By generating predictive risk scores, these tools will empower clinicians to devise personalized therapeutic strategies long before the onset of clinical relapse, ultimately leading to significant improvements in overall survival. In the near future, the clinical implementation of AI-based risk scores and the standard integration of digital twins into healthcare systems are expected to significantly transform the management of relapse risk and related clinical challenges. In this context, digital twins will serve as dynamic interfaces where individual patient risks are simulated, treatment outcomes are predicted, and these insights are directly translated into actionable clinical decisions. More than just predictions and physical evaluations, the rapidly advancing field of digital health in recent years offers strong potential to improve patients’ quality of life while substantially reducing the burden on healthcare systems. This digital health strategy will enable more efficient use of routine follow-up data after allo-HSCT, support the creation of truly personalized treatment plans, and help minimize relapse risk through better-targeted therapeutic decisions. To implement the advanced approaches envisioned in this future perspective for preventing relapse after allogeneic HSCT, large-scale prospective studies are needed to validate immune-focused preventive therapies. Addressing existing gaps through multicenter studies will be critical for translating promising monitoring strategies into evidence-based clinical practice. Importantly, the clinical translation of these technologies will depend not only on analytical performance and the generation of new knowledge, but also on cost-effectiveness, scalability, and compatibility with existing laboratory workflows.

## 8 Conclusions

Although allo-HSCT is a main curative treatment for AML, its success depends on functional IR and complex interactions between the microenvironment and leukemic blasts. Problems in this process directly increase the risk of relapse, which is the biggest barrier to survival after transplant. Today, clinical laboratories play a key role in predicting relapse using flow cytometry and MRD monitoring. By using LAIP and DfN strategies, MRD tracking helps detect genomic and phenotypic changes before clinical relapse starts. However, more research and new technologies are needed to understand the deep molecular heterogeneity and how LSCs escape from chemotherapy and immune responses. Therefore, future immune monitoring should go beyond simple cell counts and focus on cellular functional fates-like exhaustion, anergy, and senescence-and spatial interactions in the bone marrow. Combining spectral flow cytometry with single-cell multi-omics will help explain the intrinsic and extrinsic mechanisms that trigger relapse. As a complementary approach to MRD monitoring in immune surveillance of AML relapse after allo-HSCT, SERS-based liquid biopsy strategies are emerging as a promising, non-invasive technology for early relapse prediction and disease monitoring, by integrating high-sensitivity detection of serum molecular alterations with ML-based analytical frameworks. In the near future, clinical ML algorithms and digital twin technologies are expected to manage these complex data networks and create personalized risk scores. Integrating these AI tools into healthcare systems will allow digital twins to act as dynamic interfaces where individual risks are simulated and turned into real clinical decisions. This digital health strategy will make routine follow-up data more efficient, help create personalized treatment plans, and minimize relapse risk with accurate, data-driven interventions. For machine learning algorithms and digital twin frameworks to become clinically effective, the integration of heterogeneous data sources, including multi-omics, clinical variables, imaging, and longitudinal immune monitoring, requires robust data harmonization and standardization. Variability in data formats, sampling time points, and analytical pipelines currently represents a major barrier to the reliable implementation of data-driven models. Addressing these challenges through standardized data infrastructures and interoperable platforms will be essential to ensure scientific pragmatism and clinical applicability. In this context, clearly defining which immune monitoring tools are truly actionable in clinical decision-making is of critical importance. Despite impressive progress in cutting-edge immune monitoring techniques, multiparameter flow cytometry-based immune profiling and MRD assessment remain, at present, the fully validated and practically actionable methods for routine clinical use. In contrast, more approaches such as single-cell multi-omics, TCR repertoire sequencing, and spatial immune profiling provide important mechanistic insights into relapse biology but are not yet suitable for widespread clinical implementation due to high costs, lack of standardization, and the requirement for advanced bioinformatic infrastructure and expertise. Accordingly, these technologies should currently be regarded as complementary research tools rather than replacements for established clinical assays. A stepwise translational integration that bridges robust routine platforms with emerging high-level technologies will be essential to ensure that future immune monitoring strategies are both biologically informative and clinically feasible.
